# Delineating the role of nuclear receptors in colorectal cancer, a focused review

**DOI:** 10.1007/s12672-023-00808-x

**Published:** 2024-02-19

**Authors:** Mukesh Kumar Manickasamy, Sujitha Jayaprakash, Sosmitha Girisa, Aviral Kumar, Hiu Yan Lam, Elena Okina, Huiyan Eng, Mohammed S. Alqahtani, Mohamed Abbas, Gautam Sethi, Alan Prem Kumar, Ajaikumar B. Kunnumakkara

**Affiliations:** 1 Cancer Biology Laboratory, Department of Biosciences and Bioengineering, Indian Institute of Technology Guwahati (IITG), Guwahati, 781039 Assam India; 2 Department of Pharmacology, Yong Loo Lin School of Medicine, National University of Singapore, Queenstown, 117600 Singapore; 3 NUS Center for Cancer Research, Yong Loo Lin School of Medicine, National University of Singapore, Queenstown, 117699 Singapore; 4 Radiological Sciences Department, College of Applied Medical Sciences, King Khalid University, 61421 Abha, Saudi Arabia; 5 BioImaging Unit, Space Research Centre, Michael Atiyah Building, University of Leicester, Leicester, LE1 7RH UK; 6 Electrical Engineering Department, College of Engineering, King Khalid University, 61421 Abha, Saudi Arabia

**Keywords:** Colorectal cancer, Nuclear receptors, Apoptosis, Proliferation, Biomarkers, Agonists, Antagonists, Signaling pathways

## Abstract

Colorectal cancer (CRC) stands as one of the most prevalent form of cancer globally, causing a significant number of deaths, surpassing 0.9 million in the year 2020. According to GLOBOCAN 2020, CRC ranks third in incidence and second in mortality in both males and females. Despite extensive studies over the years, there is still a need to establish novel therapeutic targets to enhance the patients’ survival rate in CRC. Nuclear receptors (NRs) are ligand-activated transcription factors (TFs) that regulate numerous essential biological processes such as differentiation, development, physiology, reproduction, and cellular metabolism. Dysregulation and anomalous expression of different NRs has led to multiple alterations, such as impaired signaling cascades, mutations, and epigenetic changes, leading to various diseases, including cancer. It has been observed that differential expression of various NRs might lead to the initiation and progression of CRC, and are correlated with poor survival outcomes in CRC patients. Despite numerous studies on the mechanism and role of NRs in this cancer, it remains of significant scientific interest primarily due to the diverse functions that various NRs exhibit in regulating key hallmarks of this cancer. Thus, modulating the expression of NRs with their agonists and antagonists, based on their expression levels, holds an immense prospect in the diagnosis, prognosis, and therapeutical modalities of CRC. In this review, we primarily focus on the role and mechanism of NRs in the pathogenesis of CRC and emphasized the significance of targeting these NRs using a variety of agents, which may represent a novel and effective strategy for the prevention and treatment of this cancer.

**Graphical Abstract:**

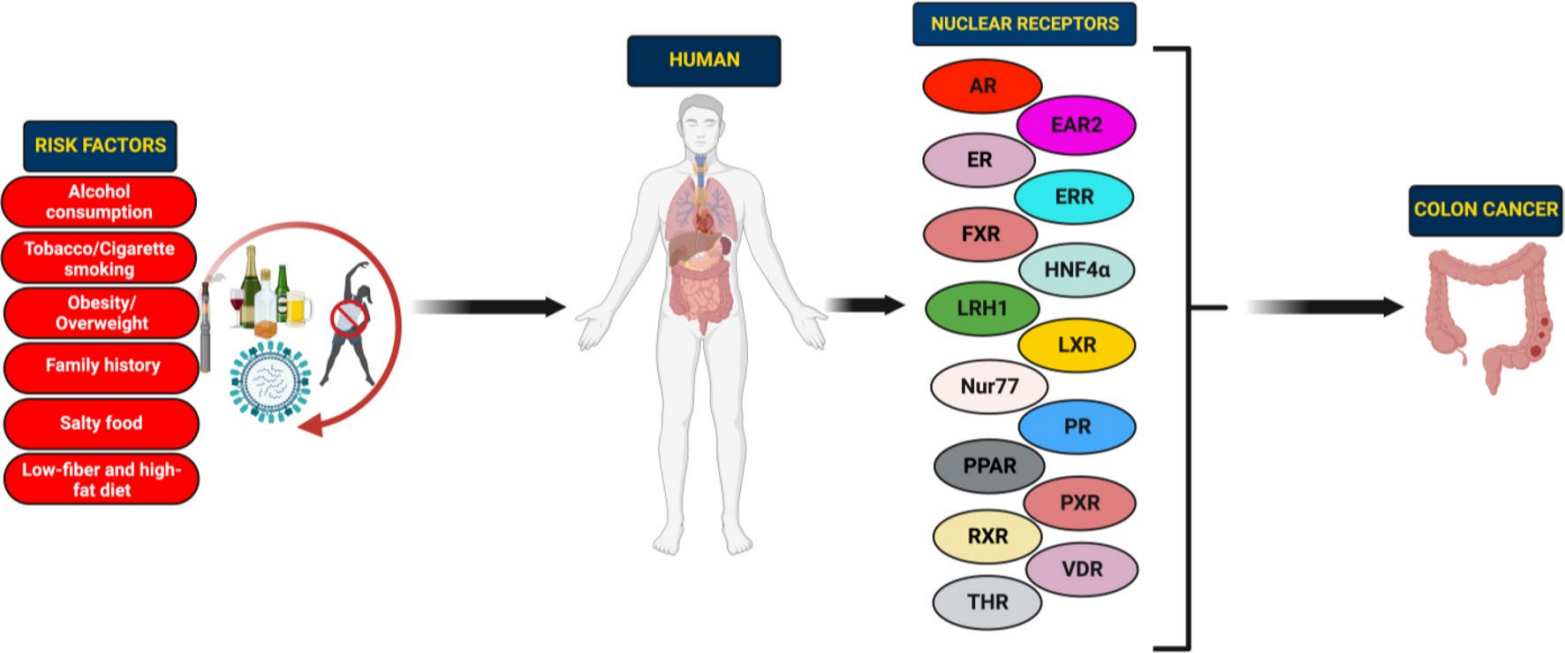

## Introduction

With more than 19.3 million new cases and 10 million fatalities recorded annually, cancer persists as one of the world’s most destructive diseases and causes of death [1]. Among these, colorectal cancer (CRC) ranks third in terms of incidence and second in fatality considering both sexes worldwide [1, 2]. Approximately 41% of all CRCs are believed to occur in the proximal colon, 22% in the distal colon, and 28% in the rectum [3]. Apparently, an increase in uptake of animal-source food, a sedentary lifestyle, less physical activity, excess body weight, and obesity are the major risk factors for the initiation, development, and progression of CRC [4]. In addition, heavy consumption of alcohol, excessive smoking, intake of red or processed meat, and various genetic and environmental aspects are also reported as key risk factors in CRC [3, 5, 6]. The detection of precancerous lesions or cancer at an early stage of CRC development has significantly risen due to the greater adoption of screening programmes [7, 8]. However, 20% of cases are identified when the illness has progressed to further organs like the liver or lung [7]. Although effective cancer screening programmes have reduced the incidence and mortality of CRC, GLOBOCAN 2020 projected 1,148,515 new cases and 576,858 deaths with respect to colon cancer and 732,210 new cases and 339,022 deaths in rectal cancer in the year 2020 [1, 9]. An approximate ninefold variation was observed in the incidence rate of colon cancer in different regions of the world, with the highest rates in New Zealand, Hungary, Europe, Norway, Australia, and Northern America [1]. Rectal cancer incidence rates are found to be the highest in the Eastern Asia region and the lowest in the regions like South-Central Asia and Africa [1]. However, the incidence rate of CRC has declined in some high-incidence countries mainly because of the healthier lifestyle choices, such as reduced consumption of tobacco, a proper dietary condition, the detection of CRC in the early stages by colonoscopy screening and the removal of precursor lesions [10, 11]. Though there are many advances made in the treatment, prevention of complications after post-operation remains a challenge in the clinical management of CRC [12, 13]. Moreover, the prognosis of CRC has never been effective, especially for patients with metastatic lesions [14]. Accumulating evidence over the past decades suggests that chemotherapy including natural compounds, have improved overall survival (OS) in cancer patients [15–28]. However, chemotherapy has been linked with a lot of drawbacks, such as systemic toxicity, unsatisfactory response rate, erratic innate and acquired resistance, and a dearth of tumor-specific selectivity [29, 30]. As a result, significant studies are required to develop innovative strategies to improve or possibly replace the current CRC chemotherapy regimen [13, 31–34]. Therefore, the exploration and identification of new therapeutic targets are imperative to enhance the management of CRC.

Nuclear receptors (NRs) are classically defined as ligand-activated transcription factors (TFs) and are divided into three groups based on their ligand-binding; orphan, adopted and the endocrine NRs [35, 36]. Currently, there are more than three hundred members in NR superfamily across the species, and forty-eight NRs are known to play a role in human physiology [35, 36]. Studies in the past have revealed the pivotal role of steroid hormones, such as androgens, in the progression of various cancers [37]. Later, numerous studies stated the critical role of NRs in cancer development and progression [38–41]. Thus, NRs have emerged as novel, highly efficacious therapeutic targets for various malignancies [35, 42–44]. It has also been observed that nuclear hormone receptors (NHRs) play a crucial role in interacting with hormonal factors in the nucleus and regulation of gene expression [36, 45]. Their varied biological and physiological properties are vast, and regulate numerous functions like differentiation, metabolism, reproduction, homeostasis, physiology, and development [36, 45]. It is well known that men are more predisposed towards colon cancer development compared to women. In line with this, accumulating evidence implicates the linkage of aberrant levels of sex hormones in the development of CRC [46]. It was shown that hormonal therapy such as dehydroepiandrosterone sulphate, an androgen precursor, improved the OS and was associated with a reduced risk in CRC patients [47]. Besides, the clinical trial by Women’s Health Initiative showed 40% decreased risk of CRC development when treated with estrogen plus progestin in the treatment cohort compared with the placebo group [48]. Further, it was reported that CAG repeats (> 25) in androgen gene in males increase their susceptibility to colon carcinogenesis, while females having CA repeats (≥ 25) in ER*β* gene had six-fold higher risk for the development of colon cancer [49]. Preclinical studies have documented the protective role of both estradiol and testosterone in colon carcinogenesis. Treatment with testosterone resulted in apoptosis of colon cancer cells via phosphatidylinositol 3-kinase/Akt (PI3K/Akt) pathway, activation of Bad, and actin cytoskeleton dynamics [50]. Amos-Landgraf and colleagues studied the sex disparity in the development of colonic adenomas in Apc^(Pirc/+)^ rat model. It was observed that ovariectomy in female rats resulted in the deprivation of endogenous hormones and had no effect on the prevalence of adenomas. However, it was reported that orchidectomy (castration) of male rats significantly protected the Apc^(Pirc/+)^ rat from adenoma development, whereas treatment with testosterone reversed this effect [51]. Though various experiments have shown the differential role of sex hormones in CRC, prospective, multi-centered, interventional clinical trials are the need of hour to successfully establish the hormonal therapy as an adjuvant to conventional cancer treatments.

**Fig. 1:**
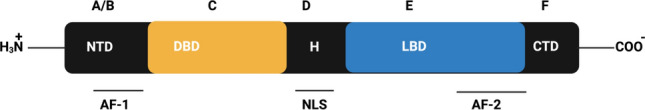
Nuclear receptor structural domains: N-terminal domain (NTD) (**A**/**B**), DNA binding domain (DBD) (**C**), hinge region (**D**), ligand/hormone binding domain (LBD) (**E**), and C-terminal domain (CTD) (**F**). NTD consists of a region, activation function-1 (AF-1), whose function is independent of the presence of a ligand. LBD consists of another region activation function-2 (AF-2), whose function is dependent on the presence of a bound ligand. Nuclear localization sequence or signal (NLS), is an amino acid sequence that tags a protein for import into the cell nucleus by nuclear transport

The NRs consists of intracellular TFs that serve as sensors for a variety of stimuli and translate the external signal into a transcriptional output [52]. All NR family members contain an N-terminal domain (NTD), a ligand binding domain (LBD), that can bind cell permeable agonists, a hinge region, a DNA-binding domain (DBD) that binds to upstream sequences of target genes, and a C-terminal domain (CTD) (Fig. 1) [52]. Dysregulation of NRs is often linked to various diseases in humans and controls complex regulatory signaling pathways in disease progression [36, 53]. The NRs REV-ERB and retinoic acid receptor-related orphan receptors (ROR) have been implicated in a wide range of physiological processes, including metabolic regulation, development, immunity, and the circadian rhythm [54]. Given that NRs govern a diverse array of biological processes that overlaps with the characteristics of cancer cells, their involvement in tumorigenesis and the advancement of cancer has been extensively studied over the decades [55]. NRs also possess a vital role in the tumor microenvironment by controlling inflammation and immune responses [56]. Furthermore, NRs also serve as biomarkers for tumor sub-classification and targets for hormone therapy which play a major role in cancer diagnosis and treatment [55, 57]. Since most NRs can be selectively activated or inactivated by small molecules, they act as prominent therapeutic targets [36]. There are several FDA-approved NR-targeted drugs for oncology use, such as flutamide, bexarotene, tamoxifen, etc. [58].

Extensive research conducted over the past decades has provided substantial insights into the role of NRs in the pathogenesis of CRC. Therefore, in this review, the influence of alterations in NRs of both normal and cancerous cellular processes in CRC is highlighted. We also demonstrate the prospect of targeting NRs as an alternate strategy for the prevention and treatment of CRC.

## Nuclear receptor signaling

Activated NRs control numerous biological processes in the body through regulating the transcription of multiple genes [59]. Recent studies have highlighted the activation of orphan NRs by various endogenous ligands, which has increased the prospect for the development of various synthetic ligands for the modulation of the NRs in the management of different diseases, including cancer [54, 60]. NRs are majorly found as monomers, but they tend to form higher-order complexes when bound to their binding partners. For example, NRs when bound to RXR, can exist as either homo or heterodimeric complexes [59, 61].

**Fig. 2:**
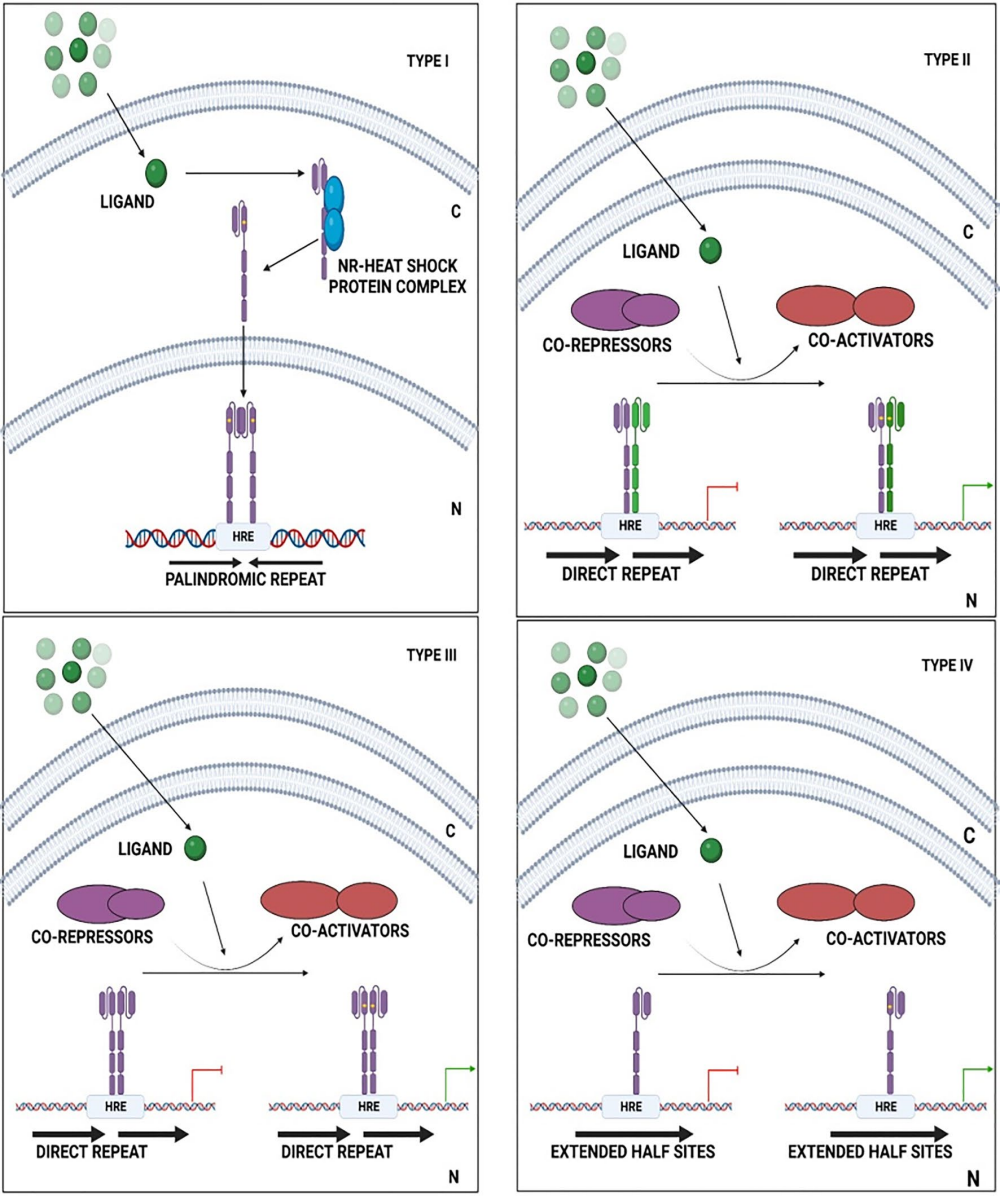
Nuclear receptor general mechanism: NRs exert their transcriptional stimulation of target genes by four different mechanisms (Type I–IV). (*N* Nucleus; *C* Cytoplasm)

Based on their modes of action, NRs signaling is divided into four types, ranging from Type I to Type IV **(**Fig. 2**)** [62]. Type I NRs: these receptors contain steroid receptors (SRs), which are activated by steroidal hormones generated from cholesterol, including androgens, estrogens, progesterone, and corticoids [61, 63]. These receptors are sequestered in the cytoplasm by chaperone proteins, but when the ligand activates, they dissociate from their chaperone proteins and translocate into the nucleus. In the nucleus, SRs often bind to DNA response elements (RE) composed of two inverted repeats as homodimers [61, 64]. Type II NRs: members of this class include RAR and LXR and are frequently found in the nucleus even without the presence of an activating ligand. Upon the binding of a ligand, the receptor undergoes a conformational change, resulting in its dissociation from a co-repressor complex. This event facilitates the subsequent recruitment of co-activators and the transcriptional machinery, initiating the process of gene transcription [61]. On direct repeat of DNA RE, these receptors typically form heterodimeric complexes with RXRs RE [59]. NRs of Type III generate homodimers on their direct repeat sequences, which are comparable to the REs of Type II NRs and have a similar mode of action [61]. Type IV NRs share a similar mode of action to that of Type II NRs, but it binds to DNA as a monomeric structure and recognizes extended half‐sites within RE [61]. NR crosstalk refers to the intricate interplay between various NRs or their intersecting signaling pathways. Some NRs, such as PPARs and RARs, participate in the formation of well-established "typical" heterodimers with RXR. Additionally, there is a distinct class of physical complexes known as the "atypical" heterodimers, wherein NRs like GR and PPARs or PPAR and ERR binds together, potentially exhibiting more transient characteristics [65]. In summary, these NRs represent viable targets for therapeutic intervention, and ligands designed to specifically target these receptors may be used as a possible strategy for the treatment of various diseases.

## Nuclear receptors in colorectal cancer

**Fig. 3:**
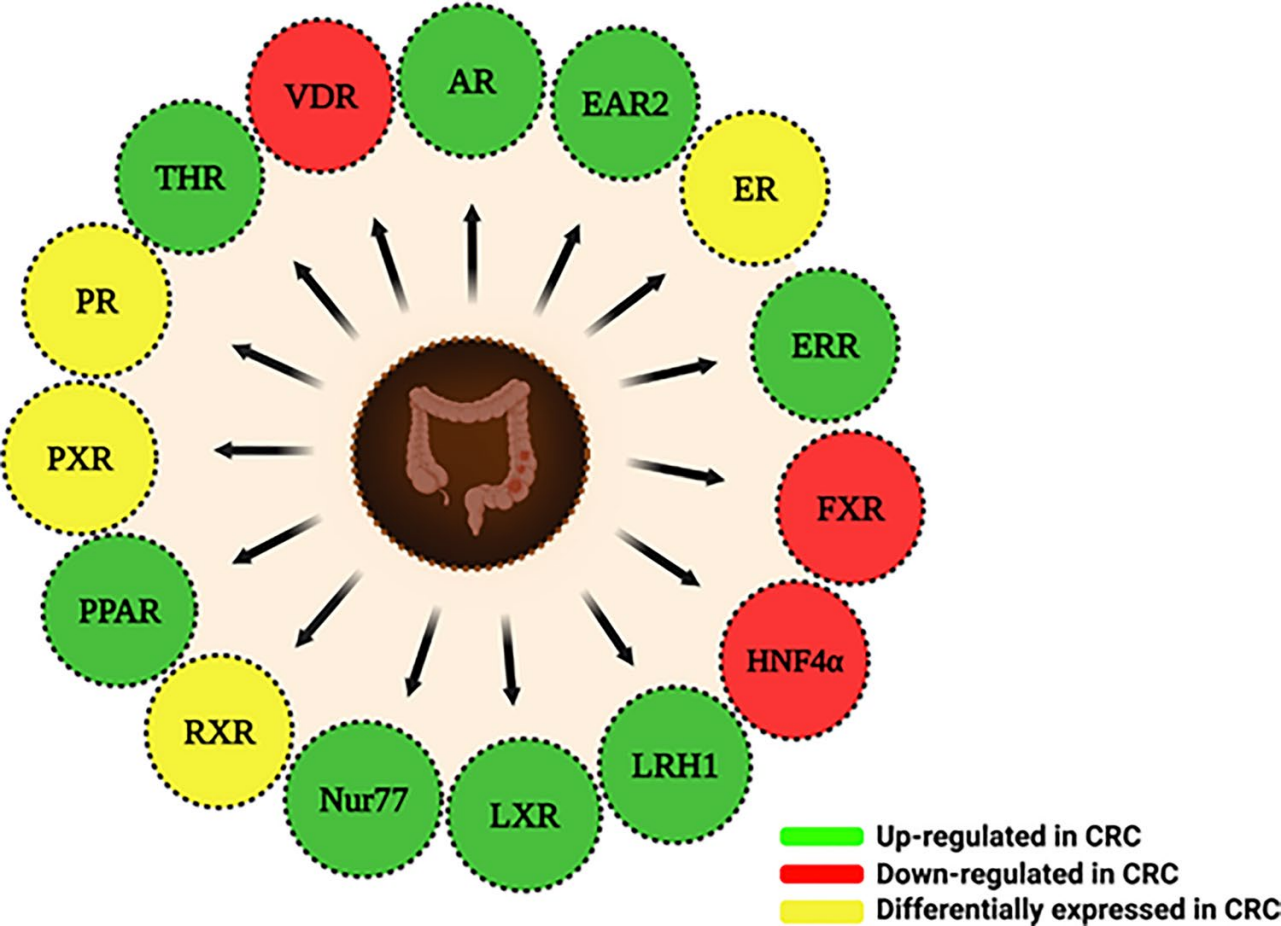
Nuclear receptors in colon cancer: Various NRs that are involved in colorectal cancer are Androgen receptors (ARs), EAR2, Estrogen receptors (ERs), Estrogen-related receptors (ERRs), Farnesoid X receptors (FXRs), Hepatocyte nuclear factor 4 alpha (HNF4*α*), Liver Receptor Homolog 1 (LRH-1), Liver X receptors (LXRs), Nuclear Hormone Receptor 77 (Nur77), Progesterone receptors (PRs), Peroxisome proliferator-activated receptors (PPARs), Pregnane X receptors (PXR), Retinoid X receptors (RXRs), Thyroid hormone receptors (THRs) and Vitamin D receptors (VDRs)

NRs have long been at the forefront of cancer research due to their ability to modulate various processes of tumorigenesis [36]. During tumor development, NRs remarkably control the growth of tumors in hormone-driven tissues [35]. Several NRs have been implicated in CRC development and progression, where they regulate multiple signaling pathways and TFs, thereby altering cellular behavior [66, 67]. The involvement of NRs in CRC has been the subject of an increasing amount of attention due to their ability to control a range of tumor-related processes, including cell proliferation, differentiation, homeostasis, migration, invasion, and cell death (Table 1 and 2). The significant role of several NRs such as Androgen Receptors (ARs), EAR2, Estrogen Receptors (ERs), Estrogen-related Receptors (ERRs), Farnesoid X Receptors (FXRs), Hepatocyte Nuclear Factor 4 alpha (HNF4*α*), Liver Receptor Homolog 1 (LRH-1), Liver X Receptors (LXRs), Nuclear Hormone Receptor 77 (Nur77), Progesterone Receptors (PRs), Peroxisome Proliferator-activated Receptors (PPARs), Pregnane X Receptors (PXR), Retinoid X Receptors (RXRs), Thyroid Hormone Receptors (THRs) and Vitamin D Receptors (VDRs) has been identified in CRC (Fig. 3). Through the regulation of various TFs and signaling cascades, these receptors help in the development and progression of CRC.

**Table 1:** Nuclear receptors expression in colon cancer and various CRC cell lines

Nuclear receptor	In vitro/In vivo/In situ/Clinical	Cell lines/tissue/animal models	Regulation	Oncogenic/tumor-suppressive	References
AR-B	Clinical	CRC tissues	Down	Tumor suppressive	[71]
AR	Clinical	CRC tissues	Up	Oncogenic	[72]
	Clinical	CRC tissues	Down	Tumor suppressive	[73]
	Clinical	CRC tissues	Down	Tumor suppressive	[74]
EAR2	Clinical	Primary CRC tissues	Up	Oncogenic	[75]
ER	Clinical	CRC tissues	Up	Oncogenic	[76]
ER-*α*46	Clinical	CRC tissues	Down	Tumor suppressive	[78]
ER*α*	Clinical	CRC tissues (Men)	Up	Oncogenic	[77]
	In situ	CRC tissues	Up	Oncogenic	[79]
	In vivo	F344 Rats	Down	Tumor suppressive	[80]
	In vivo	Mice	Up	Oncogenic	[81]
	Clinical	CRC tissues	Up	Oncogenic	[82]
ER*β*	Clinical	CRC tissues	Down	Tumor suppressive	[77]
	In situ	CRC tissues	Up	Oncogenic	[79]
	Clinical	CRC tissues	Down	Tumor suppressive	[82]
	Clinical	CRC tissues	Down	Tumor suppressive	[83]
	Clinical	CRC tissues	Up	Oncogenic	[84]
	In vitro	SW480, SW620, HT-29	Up	Oncogenic	[84]
	Clinical	CRC tissues	Up	Oncogenic	[85]
	Clinical	CRC tissues	Down	Tumor suppressive	[86]
	Clinical	CRC tissues	Down	Tumor suppressive	[87]
	Clinical	CRC tissues	Down	Tumor suppressive	[88]
	Clinical	CRC tissues	Down	Tumor suppressive	[89]
	Clinical	CRC tissues	Down	Tumor suppressive	[90]
	Clinical	CRC tissues	Down	Tumor suppressive	[91]
	Clinical	CRC tissues	Down	Tumor suppressive	[92]
	In vivo	Mouse model	Down	Tumor suppressive	[93]
	Clinical	CRC tissues	Up	Oncogenic	[94]
	Clinical	Colon cancer tissues	Down	Tumor suppressive	[95]
ERR*α*	Clinical	CRC tissues	Up	Oncogenic	[96]
	Clinical	Colon cancer tissues	Up	Oncogenic	[97]
	In vitro	HCT-116p53^+/+^, Lim1215, HT-29, DLD-1, HCT-15, HCT-116p53^−/−^	Up	Oncogenic	[98]
	Clinical	CRC tissues	Up	Oncogenic	[99]
ERR*γ*	In vitro	HCT-116p53^+/+^, Lim1215, HT-29, DLD-1, HCT-15, HCT-116p53^−/−^	Down	Oncogenic	[98]
FXR	Clinical	Human intestinal mucosa section	Down	Tumor suppressive	[100]
	Clinical	CRC surgical specimens	Down	Tumor suppressive	[101]
HNF4*α*	Clinical	Colon carcinoma tissues	Down	Tumor suppressive	[102]
LRH-1	Clinical	Colon cancer tissues	Up	Oncogenic	[103]
	Clinical	CRC tissues	Up	Oncogenic	[104]
	In vitro	HT-29, HCT-116, SW480, SW620	Up	Oncogenic	[105]
LXR*α*	Clinical	CRC tissues	Down	Tumor suppressive	[106]
Nur77	Clinical	CRC tissues	Up	Oncogenic	[107]
	Clinical	CRC tissues	Up	Oncogenic	[108]
PPAR	In vivo	PPAR*β* ^+/+^ and Apc^+/−^ AOM-treated mice	Down	Tumor suppressive	[109]
	Clinical	CRC tissues	Down	Tumor suppressive	[110]
	Clinical	CRC tissues	Down	Tumor suppressive	[111]
PPAR*δ*	In vivo	C57BL/6 J APC ^min/+^	Up	Oncogenic	[112]
	Clinical	CRC tissues	Up	Oncogenic	[113]
PPAR*δ*/*β*	In vitro	Mouse lines [villin-PPAR-d-1 and villin-PPAR-d-2]	Up	Oncogenic	[114]
PPAR*γ*	Clinical	Colon adenocarcinomas tissues	Down	Tumor suppressive	[115]
	Clinical	Colon cancer tissues	Up	Oncogenic	[111]
	Clinical	Colon cancer tissues	Up	Oncogenic	[116]
	In vivo	AOM-induced F344 rats	Up	Oncogenic	[117]
	In vitro	HT-29, HCT-116, KM12, HCC2998	Up	Oncogenic	[118]
PXR	In vitro	HT-29	Down	Tumor suppressive	[119]
	Clinical	CRC tissues	Down	Tumor suppressive	[120]
	In vitro	LS174T, HT-29, HCT-116, SW480, SW620	Down	Tumor suppressive	[120]
	Clinical	CRC tissues	Up	Oncogenic	[121]
PR	Clinical	Rectal cancer tissues	Up	Oncogenic	[122]
	Clinical	CRC tissues	Up	Oncogenic	[76]
	Clinical	CRC tissues	Down	Tumor suppressive	[123]
RXR*α*	In vitro	Caco2, HT-29, Colo201, Colo320, DLD‐1, HCT‐116, SW837	Up	Oncogenic	[124]
	Clinical	CRC tissues	Up	Oncogenic	[125]
	In vitro	HT-29, SW 480	Up	Oncogenic	[125]
	In vivo	Rat tissues	Down	Tumor suppressive	[126]
	Clinical	CRC tissues	Down	Tumor suppressive	[127]
THR	Clinical	CRC tissues	Up	Oncogenic	[128]
	Clinical	CRC tissue	Down	Tumor suppressive	[129]
VDR	In vivo	Apc^Min/+^ mice	Down	Tumor suppressive	[130]
	In vivo	Mouse model	Down	Tumor suppressive	[131]
	In vivo	Mice (C57BL/6 J)	Down	Tumor suppressive	[132]
	In silico	Data from LS180 colon cancer cells	Down	Tumor suppressive	[133]
	Clinical	CRC tissues	Up	Oncogenic	[134]
	Clinical	Ulcerative colitis-CRC tissues	Down	Tumor suppressive	[135]
	Clinical	Colon tissues	Up	Oncogenic	[136]
	Clinical	CRC tissues	Down	Tumor suppressive	[137]
	Clinical	CRC tissues	Down	Tumor suppressive	[138]

**Table 2:** Mechanistic role of various nuclear receptors in colon cancer in the presence of their agonists/antagonists

Nuclear receptor	In silico/In vitro/In vivo/Clinical	Cell lines/tissue/animal models	Agonist/antagonist	Expression	References
AR	In vitro	HCT-8, HT-29	FCX	↓ Cell growth	[139]
	In vivo	MC-38 xenograft model	Enzalutamide	↑ Tumor growth	[140]
	In vitro	MC-38	Enzalutamide	↓ Cell number	[140]
	In vitro	Caco2	DHEA, NGF Testosterone	↓ Apoptosis	[50]
				↑ TrkA receptor	
	In vitro	SW480	DHT	↑ G_1_ cell cycle arrest	[141]
				↓ Cell growth, TOPFLASH activity	
	In vivo	C-26 model	SARM and HDACi (GTx‐02, AR-42)	Anti‐cachectic effects	[142]
				↑ Body weight, hindlimb skeletal muscle mass, Grip strength, Survival	
	In vivo	C-26 model	HDACi (AR-42)	↓ IL-6/GP130/STAT3	[142]
EAR 2	In vitro	HCT-116, RKO, HT-29	siEAR2	↑ Apoptosis ↓ XIAP	[75]
	In vivo	Nude mice (EAR2 inactivated RKO cells xenograft)	shEAR2	↑ Apoptosis ↓ Tumor growth	[75]
ER	In vitro	HCT-116, HCT8	Raloxifene	↓ Cell proliferation	[143]
	In vitro	HCT8	Tamoxifen	↓ Cell growth	[143]
	In vitro	YAMC	E2, Apigenin, Naringenin	↓ Cell growth	[144]
	In vitro	HCT-116, DLD1	17βE2	↓ Cell number	[145]
	In vitro	HCT-8	17βE2	↑ Cell number	[145]
	In vitro	SW480	Lentivirus ER*β* OE	↓ c-Myc, cyclin E, p45Skp2, pRB, pRB-Ser795	[86]
				↑ cyclin D, p14ARF, p21Cip, p27Kip1, pRB-Ser 780, p53	
				G1 cell cycle arrest	
	In vitro	HCT-116	Lentivirus ER*β* OE	↓ Proliferation, c-Myc, p21Cip, p53	[86]
	In vitro	HT-29	Lentivirus ER*β* OE	↑ p21Cip, p27Kip1, p53	[86]
				↓ c-Myc	
	In vivo	SCID mice (SW480-ER*β* cells)		↓ Ki67-positive cells, Tumor weight	[86]
	In vitro	HCT-116	Raloxifene	↓ Cell growth, PCNA, ER*β*	[146]
	In vivo	AOM induced mode in F344 rats	Raloxifene	↓ Body weight at 5ppm conc, ACF formation	[146]
	In vitro	MC38	Diarylpropionitrile	↓ Cell growth	[88]
	In vitro	SW480, HCT-116	ERB-041	↑ ESR2, CYSLTR2, HPGD, CCND1	[147]
				↓ Migration, Colony formation, CYSLTR1, PTGS2, CTNNB1, Myc, Survival	
	In vitro	Caco2	ERB-041	↑ ESR2, CYSLTR2, HPGD, p-*β-*catenin*, β-*catenin	[147]
				↓ CYSLTR1, PTGS2, CTNNB1, CCND1, Myc	
	In vitro	SW480	ERB-041	↓ Migration, Survival	[147]
	In vivo	HT-29 zebrafish xenograft model	ERB-041	↓ Cell metastasis	[147]
	In vitro	LoVo	ER*α* (OE)	↑ DNA fragmentation, Caspase 3, -8, -9	[148]
	In vitro	LoVo	ER*α* (OE) + E2	↑ hTNF-*α*, DNA fragmentation, p21, p27, Apoptosis, Caspase -3, -8, -9	[148]
				↓ β*-*catenin, cyclin D1, Rb, Proliferation, Metastasis	
	In vivo	ER*β* KO Mice	E2	↑ Apoptosis	[81]
	In vivo	C57BI6/J Mice	E2	↓ Number and average area of adenocarcinomas, Apoptosis	[81]
				↑ Cell proliferation	
	In vitro	Deficient hMLH1- HCT-116(re-expression of hMLH1)	Estradiol	↓ Cell viability	[149]
				↑ Apoptosis, Caspase-3, -9, Bax, p53	
	In vitro	hMLH1 overexpression in LoVo	Estradiol	↑ Apoptosis	[149]
	In vitro	HCT-116	ER*β* OE (EGFP-C1 and EGFP-C1-Er*β*)	↓ Cell growth, Proliferation, cyclin D1, mTOR	[150]
				↑ Autophagy, BNIP3	
	In vitro	DLD-1	Estradiol, 17 Epiestriol, Quercetin with Tamoxifen	↓ Cell growth, Thymidilate synthase/G6PDH, Survivin	[151]
	In vitro	DLD-1, HT-29	Estradiol (Hypoxic condition)	↑ Migration, Proliferation, Wound closure	[152]
	In vitro	DLD-1, HT-29	Estradiol (Normoxia condition)	↓ Migration, Proliferation	[152]
				↓ Wound closure, Cell growth	
	In vitro	DLD-1	Soy isoflavones	↑ ER*β*	[153]
	In vivo	Male and female Sprague–Dawley rats	Soy isoflavones	↓ Tumor dysplasia	[153]
				↑ ER*β*	
	In vivo	ER*β*^*−*/−^APC^min/+^	–	↑ Tumor area, Polyp size	[154]
	In vivo	C57BL/6 J mice	E2	↑ TGFB1, TGFB2, TGFB3, INHBE, RUNX1	[154]
	In vitro	COLO205	E2	↑ Apoptosis, ER*β*, MLH1	[155]
				↓ miR-31, miR-155, miR-135b	
	In vitro	HT-29	Tamoxifen, 5-FU	Block the cells in G2/M phase	[156]
				↓ Cell proliferation, Migration, MMP-7, ER*β*	
				↑ Apoptosis	
	In vitro	DLD-1	17*β*-E2	↑ ER*β*, p38/MAPK	[157]
	In vitro	COLO-205	E2	↑ Apoptosis, Dose dependent DNA fragmentation, Nuclear condensation	[158]
	In vivo	AOM induced model	Celecoxib + DFMO	↑ ER*α*, DNA methylation	[80]
	In vitro	DLD-1	17*β*-E2	↑ NGB, Apoptosis	[159]
	In vivo	Sprague–Dawley rats	E2	↑ ER*α*, Mitotic index	[160]
	In vitro	DLD-1 cells	Quercetin	↓ Cell number	[161]
				↑ Caspase 3 activation, Cleaved PARP, p-p38	
	In vitro	HT-29 cells	5-Aza-CdrR	↓ Cell growth, DNMT1	[162]
				↑ Apoptosis, ER*α*	
	In vitro	SW480, HT-29	ER*β*	↑ TNF-*α*	[93]
	In vivo	ER*β*KO^Vil^ mice (Females) (AOM/DSS)	–	↑ IL-6, Cc12, Cc14, IL-1B, TNF-*α*, No. of tumors	[93]
	In vivo	ER*β*KO^Vil^ mice (Males) (AOM/DSS)	–	↑ No. of tumors, IL-6, Cc12, Cc14, TNF-*α*	[93]
				↓ IL-1B	
	In vivo	Sprague–Dawley rats	E2 + P4	↑ Apoptotic index, Caspase 3 score, Cleaved PARP, Cleaved caspase 8, ER*β*	[160]
				↓ M/A ratio, PCNA score	
	In vitro	SW480	Cisplatin	↑ Cell viability	[163]
	In silico	Colon tissues	–	↓ miR-205	[95]
				↑ PROX1	
	In vitro	SW480, HT-29	miR-205	↑ Adhesion	[95]
				↓ Tumor invasion, Proliferation	
	In vivo	SW480 Xenograft (Zebra fish)	ER* β* and miR-205	↓ Tumor Invasion	[95]
ER-*α*46	In vitro	ER*α*46-transfected cells HT-29	17*β*-estradiol	Accumulation of cells in the G (0/1) phase	[78]
				↓ Cell growth, proportion of cells in G (2)/M phase	
				↑ Apoptosis	
ERR	In vitro	HCT-116, HT-29, DLD1	shERR*α*	↓ Cell growth, Colony formation, CDK2, cyclin D1, E2F4, E2F1, Cdc25A, cyclin A, c-Myc, pRb	[164]
				↑ G1/S checkpoint arrest, p27 ^Kip1^, p15 ^INK4B^	
	In vitro	HCT-116	shERR*α*	↓ HK1, G6PC, PFKFB1, PFKFB2, ALDOC, GPT2, PGM2, Got1, Aldh4a1	[164]
	In vitro	HT-29	shERR*α*	↓ Lactate production	[164]
	In vivo	Nude mice (HCT-116) xenografts	shERR*α*	↓ Tumor growth rate, Tumor volume, Tumor weight	[164]
	In vitro	HT-29	shERR*α*	↓ OPN	[96]
	In vitro	HCT-116, SW480	shERR*α*	↓ Cell growth, Cell proliferation, Colony information	[97]
	In vitro	HCT-116, SW480, SW1116	XCT790	↓ ERR*α*, Cell growth, Cell proliferation, c-Myc, cyclin D1, Migration	[97]
	In vitro	HCT-116, SW480, SW1116	EGF	↑ ERR*α*, p-ERK, c-Myc	[97]
	In vitro	HCT-116, SW480	Trametinib	↓ ERR*α*, Cell growth, IDH3A, c-Myc, cyclin D1	[97]
	In vitro	HCT-116, SW480	Simvastatin	↓ Cell proliferation, Colony formation, IDH3A, c-Myc, cyclin D1	[97]
	In vitro	HCT-116, SW480	Trametinib + Simvastatin	↓ Cell survival, Cell proliferation, ERR*α*, IDH3A, c-Myc, cyclin D1	[97]
				↑ Bax	
	In vivo	Nude mice (HCT-116) xenograft	Trametinib + Simvastatin	↓ Tumor volume, Tumor weight, ERR*α*, IDH3A, c-Myc, cyclin D1	[97]
	In vitro	HCT-116 p53^+/+^, DLD-1, or HCT-15	shERRα	↓ p53	[98]
	In vitro	HCT-116 p53^+/+^	shERR*α*	↓ mtOxPhos, PGC1*α*, mtDNA copy number, COX-4, VDAC1, Intracellular ATP, Cell proliferation	[98]
	In vitro	HCT-116 p53^+/+^, Lim1215, HT-29, DLD-1, HCT-15, SW480, WiDr, HCT-116 p53^−/−^, Caco2	XCT790	↓ Cell proliferation, p53	[98]
	In vitro	HCT-116 p53^+/+^	XCT790	↓ COX-4, VDAC1	[98]
	In vivo	SCID mice (patient-derived colon tumor explants)	XCT790	↓ Tumor volume, Tumor weight	[98]
FXR	In vivo	2,4,6-trinitrobenzene sulfonic acid-induced colitis mice model	Probiotics (VSL3)	↑ FXR, PPAR*γ*, PXR	[165]
				↓TNF-*α*, IL-6, IFN*γ*	
	In vitro	HT-29, Caco2, HCT-116	siFXR	↑ Wnt/*β*-catenin, *β*-catenin/TCF4	[166]
	In vitro	HT-29, Caco2, HCT-116	GW4064	↓ Cell proliferation	[166]
	In vivo	AOM/DSS model	–	↓ FXR, ↑ *β*-catenin	[166]
	In vivo	FXR knockout mice	–	↑ Cell proliferation, IL-6, cyclin-D1, Adenoma size	[167]
	In vitro	SNU-C4	GW4064	↓ Cell proliferation, p-EGFR, p-Src (Tyr416), p-ERK1/2	[168]
	In vitro	HT-29	Guggulsterone	↑ Cell proliferation, p-EGFR, p-Src (Tyr416), p-ERK1/2	[168]
	In vitro	SNU-C4	FXR siRNA	↑ p-EGFR, p-Src (Tyr416), p-ERK1/2, Cell proliferation	[168]
	In vitro	HT-29	pcDNA3.1hFXR	↓ Cell proliferation, p-EGFR	[168]
	In vivo	HT-29 xenograft	hFXR OE	↓ Tumor growth	[168]
	In vitro	HCT-116	Chenodeoxycholic acid (CDCA)	↑ miR-22, FGF19	[169]
				↓ CCNA2	
	In vitro	HCT-116	GW4064	↑ miR-22, FGF19	[169]
	In vivo	C57BL/6 WT Mice	FXR KO	↑ CCNA2, Ki-67 positive cells	[169]
				↓ miR-22	
	In vitro	HCT-116, SW480, DLD-1	GW4064	↑ DR5, FXR	[170]
	In vitro	HCT-116, SW480, DLD-1	GW4064 + TRAIL	↓ Cell proliferation	[170]
	Clinical	Human intestinal mucosa section	–	↓ FXR	[100]
	In vitro	HCT-116	APC knockdown	↑ c-Myc, ↓ FXR expression	[171]
	In vivo	APC^min/+^ mice	APC knockdown	↓ FXR, SHP, IBABP	[171]
				↑ COX-2	
	In vitro	Caco2, HT-29, SW620, SW480	CDCA, GW4064	↑ IBABP, ↓ FXR	[101]
	In vitro	SW620, HCT-116	GW4064	↑ CCNG2, Cell death	[172]
				↓ miR-135A1	
	In vitro	SW620, HCT-116	FXR siRNA	↑ miR-135A1, ↓ CCNG2	[172]
	In vivo	FXR^±^Apc^Min/+^ C57BL/6 mice	–	↑ Mortality, No. of tumor per mouse	[173]
	In vitro	HT-29	pCDNA3.1hFXR	↓ MMP-7, Cell proliferation	[174]
	In vitro	HT-29	CDCA, GW4064	↓ MMP-7	[174]
	In vitro	HT-29	Guggulsterone	↑ MMP-7	[174]
	In vitro	MC38	6E-CDCA	↓ Cell invasion	[174]
	In vivo	FXR knockout mice (B6.129X1 (FVB)-Nr1h4tm 1Goz/J)	–	↑ MMP-7	[174]
HNF4*α*	In vitro	SW480, HCT-116	LINC00858 (OE)	↑ HNF4*α*, Cell Proliferation, Invasion, Migration, Angiogenesis	[175]
				↓ WNK2	
	In vivo	Nude mice	LINC00858 (OE)	↑ Tumor growth, Angiogenesis	[175]
				↓ WNK2	
	In vitro	HT-29, LoVo, SW480 cells	HNF4 *α* (OE Lentivirus)	↓ Migration, Invasion, Proliferation, Wnt-*β*-catenin ↑ G2/M Phase Arrest, Apoptosis	[102]
	In vivo	SW480 mice xenograft	HNF4*α* (OE Lentivirus)	↓ Tumor growth, Liver metastasis, Snail, Slug, Twist	[102]
	In vitro	HCT-116	HNF4*α*2	↑ Growth suppression, Cell death	[176]
	In vitro	HCT-116	HNF4*α*8	↑ Cell proliferation, Anti-apoptosis	[176]
	In vivo	Athymic nude male mice (HCT-116)	HNF4*α*2	↓ Tumor weight	[176]
	In vivo	Athymic nude male mice (HCT-116)	HNF4*α*8	↑ Tumor weight	[176]
	In vitro	HM7 cells	siRNA	↓ Proliferation, Differentiation, MUC 4	[177]
LRH-1	In vitro	HCT-116, HT-29	GATA6	↑ LRH-1	[178]
		LRH-1 overexpressing clones of HCT‐116 (OED and OEJ) and HT‐29 (OE7 and OE8)	GATA6	*↑* CD44*, *CD133*, *LGR5*,* ALDH‐1, Ascl2, Oct4, Klf4, Nanog, Sox2, CD133^+^/CD44^+^ subpopulations	[178]
	In vitro	LRH‐1‐overexpressing HCT‐116 (OED) and HT‐29 (OE7) clones	GATA6	↑ HIF‐1*α*, glycolysis, Glut-1, LDHA, PDK-1, MCT-4	[178]
	In vitro	116 Vec, OED, OEJ and 29Vec, OE7, OE8 clones	GATA6	↑ ROS, mitochondrial respiration, NDUFB8 (complex I), SDHB (complex II), UQCRC2 (complex III), MTCO1 (complex IV), ATP5A (complex V)	[178]
	In vitro	HCT-116, SW480	OE miR-374b	↓ LRH-1, Proliferation, Invasion, Wnt signaling	[105]
	In vitro	Caco2, HT-29	shRNA	↓ Proliferation, Wnt5A, ApoA4, ApoC3, SLC10A1, AKR1D1	[179]
				↑G0/G1 phase	
	In vitro	SW480, HCT-116	OE miR-136	↓ LRH-1, Proliferation, Invasion, Wnt signaling, cyclin D1, cyclin E1, c-Myc, Axin2	[180]
	In vitro	SW480, HCT-116	miR-381 antisense oligos (↓miR-381)	↑ Cell proliferation, Invasion, LRH-1	[181]
	In vivo	Male BALB/c nude mice (SW480 cells stably expressing miR-381 antisense oligos)	miR-381 antisense oligos (↓miR-381)	↑ Tumor growth	[181]
	In vitro	HT-29	1-(3′-(1-[2-(4-morpholinyl)ethyl]-1H-pyrazol-3-yl)-3-biphenylyl)ethanone dihydrochloride	↓ Proliferation	[182]
	In vitro	HCT-116, HT-29	OE miR-203	↓ Migration, Anchorage‐independent growth ability, Colony formation	[183]
	In vitro	HT-29	OE miR-203	↓ Klf4 levels, Oct 4, Nanog	[183]
	In vivo	BALB/c nude mice (GATA6‐OE HT‐29 cells)	–	↑ Tumor volume, Tumor weight	[183]
	In vitro	GATA6‐overexpressing HCT‐116	LXR, Hes-1 knockdown	↓ Sphere forming abilities	[183]
	In vitro	LoVo, SW480	OE miR-30d	↓ Cell migration, Invasion, cell cycle arrest, Cell proliferation	[104]
				↑ Apoptosis	
	In vivo	BALB/c male nude mice (SW480 cells with miR-30d)	OE miR-30d	↓ Tumor growth	[104]
	In vivo	C57BL/6 J mice	Lrh-1^+/−^ mice	↓ Tumor multiplicity	[184]
				↑ TNF-*α*	
	In vitro	Caco2	shLRH-1	↓ Cortisol synthesis and release, PPAR*γ*	[185]
	In vitro	HCT-116	siRNA	↓ Cell growth	[186]
				↑ CCNB1, CCNB2, CCNE2, p21	
LXR	In vitro	HT-29 CD133^+^	SR9243	↓ PFKB3, GSK3β, SCD-1, FASN, HIF-1α, LXR, Colony formation	[187]
				↑ ROS levels	
	In vitro	HT-29 CD133^+^	T0901317	↑ ABCA1, ABCG5, ABCG8, LXR	[187]
				↓ Ki-67, Migration, Invasion	
	In vitro	HT-29 CD133^+^	T0901317, SR9243	↑ Apoptosis	[187]
	In vitro	HCT-116	T0901317	↑ Cell death, Caspase-1	[188]
	In silico	Colon adenocarcinoma tissues	–	↓ Alpha-2-macroglobulin, afamin, albumin, apolipoprotein A-I, apolipoprotein A-II, apolipoprotein C-III, apolipoprotein H, ceruloplasmin, decorin, group-specific component/vitamin D binding protein, hemopexin, orosomucoid 1, orosomucoid 2, serpin peptidase inhibitor, clade A, member 1, serpin peptidase inhibitor, clade F, member 1, transferrin, Vimentin	[189]
	In vitro	HCT-116, HT-29, HCT8, SW480	T0901317	↑ Cell death	[190]
	In vitro	HCT-116	T0901317	↑ an early caspase-1 activation (within the first hour of treatment) and a late caspase-7 activation	[190]
	In vivo	Balb/c xenograft (CT-26)	T0901317	↓ Tumor growth	[190]
	In vitro	Colo205	GW3965	↑ ABCA1/G1, SREBP1c, SCD1, cyclin-D1, LXR, G1/G0 phase	[191]
				↓ S-phase, Cell viability, Cell proliferation, CDK2, CDK4, cyclin E, cyclin B1, CDK1, Skp2, c-Myc, Rb	
	In vitro	Colo205, HCT-116	shLXRalpha/LXRbeta	↑ Cell proliferation	[191]
				↓ % Cells in G1-G0 phase	
	In vivo	LXR*αβ*-/- mice	GW3965	↓ Ki67, Proliferation	[191]
	In vitro	HT-29	GW3965	↑ Cells in G1 phase, p21*,* Apoptosis	[106]
				↓ Cell growth	
	In vivo	C57BL/6/APC^Min/+^ mice	GW3965	↑ Caspase-Dependent Apoptosis	[106]
				↓ Tumor growth, Tumorigenesis	
	In vitro	HCT-116	T0901317	↓ TOPGLOW activity, *β*-catenin, Cell proliferation, Myc, MMP-7, BMP4	[192]
				↑ ABCA1	
	In vitro	HCT-116	GW3965	↓ TOPGLOW activity	[192]
	In vitro	HCT-116	LXR623	↑ Apoptosis	[193]
	In vitro	HCT-116	LXR623, ABT263	↓ Cellular viability	[193]
	In vitro	HCT-116	LXR623, BH3	↑ Apoptosis	[193]
	In vivo	CrTac:NCr‐Foxn1 nu (xenograft)	LXR623, ABT263	↓ Tumor volume	[193]
				↑ Apoptosis, TUNEL- positive cells	
	In vitro	HCT-116, CT26	T0901317	↑ Apoptosis, Pyroptosis, CRT, p-eIF2*α*, HMGB-1 release	[194]
	In vivo	Balb/c mice (CT26 cells) xenografts	T0901317	↓ Tumor growth, Tumor volume	[194]
				↑ CRT, HMGB-1	
	In vivo	Balb/c mice (CT26 cells) xenografts	T0901317 + HMGB-1, CRT	↓ CRT, HMGB-1	[194]
				↑ Tumor growth, Tumor volume	
	In vitro	Caco2, SW620	27-OHC	↓ Cell proliferation, Cell viability, p-Akt	[195]
	In vitro	Caco2, SW620	GW3965	↑ ABCA1, ABCG1	[195]
Nur77	In vitro	HCT-116	RA	↑ miR-22, Nur77, Apoptosis, RAR* β*	[196]
	In vivo	HCT-116 Xenograft mice (Athymic nude mice)	RA	↑ miR-22, Nur77, Apoptosis, RAR* β*	[196]
				↓ Tumor growth/Size	
	In vitro	RKO	C-DIMs	↑ TRAIL	[107]
		RKO, HCT-116, HT-29	DIM-C-pPhOCH3, DIM-C-Ph	↓ Cell growth, Proliferation	
		RKO, SW480, HCT-116, HT-29		↑ Apoptosis, Cleaved PARP, Cleaved caspase -3, -8, -9, PDCD1, CSE, ATF3	
			DIM-C-pPhOCH3	↑ PDCD1, ATF3, CSE	
			DIM-C-pPhOCH3	↑ Apoptosis, c-PARP	
	In vivo	Athymic nude mice (RKO cell) xenografts	DIM-C-pPhOCH3	↓ Tumor volume, Tumor weight	[107]
	In vitro	SW620 HCT-116	DCA siRNA DCA	↑ Nur77, Cell growth, BRE mRNA, VEGF, p-Akt, JNK, p–c-Jun, c-Fos, *β*-catenin	[108]
				↓ BRE mRNA, VEGF	
				↑ Nur77, Colony formation	
				↓ Colony formation	
				↑ Apoptosis	
	In vivo	Male Kunming mice	DMH DCA	↑ Nur77	[108]
				↑ Nur77, PCNA	
	In vitro	RKO	DIM-C-pPhOCH3	↑ Cell death, Apoptosis, TRAIL	[197]
				↓ Colon cancer cell growth	
	In vivo	RKO cell Xenograft (Athymic mice)	DIM-C-pPhOCH3	↑ NAG-1, Apoptosis, Phosphorylation of c-Jun N-terminal kinase, CHOP, DR5	[197]
	In vitro	SW480, HC-116, HT-29	(Hypoxia)	↑ *β*-catenin, Nur77, Colon cancer cell growth, Migration, Invasion, EMT	[198]
	In vitro	RKO, SW480	Knockdown of NR4A1 (SiNR4A1)	↑ Apoptosis, Cell proliferation	[199]
	In vitro	RKO	DIM-C-pPhOH and knockdown of NR4A1	↓ mTOR, Sp1	[199]
				↑ p53/sestrin2/AMPK*α*	
	In vitro	RKO and SW480	DIM-C-pPhOH	↓ Cell growth	[199]
				↑ Apoptosis	
	In vitro	HCT-15	Indomethacin	↑ Nur77, Apoptosis	[200]
	In vitro	HCT-15 (pre-treated with Retinoids)	Indomethacin	↓ Nur77	[200]
	In vitro	RKO, SW480 (Knockdown of NR4A1 by RNAi)	DIM-C-pPhOH and DIM-C-pPhCO2Me	↓ Migration, *β*1-integrin expression, Adhesion	[201]
	In vitro	HCT-116, HT-29	DCA, LCA, CA	↑ Nur77, TNF-*α*, NF*κ*B, c-FOS, c-Jun, ATM, LIG4, TP53, Apoptosis, Cell growth	[202]
	In vitro	RKO, SW480	C-DIMs	↑ Nur77, PPAR*γ*, CHOP, Death receptor 5, Cleavage of caspase 8, PARP, Apoptosis, JNK pathway	[203]
	In vivo	Mice (RKO cells xenograft)	DIM-C-pPhBr	↓ Tumor size, Tumor weight	[203]
				↑ Apoptosis, p-JNK	
	In vitro	LS-174 T, HCT-116	Cyclooxygenase-2-derived prostaglandin E2	↑ NR4A2, PPRE activity	[204]
	In vivo	DMH-induced CRC rats	Diac and/or 5-FU	↓ IL6, K-*ras*, NICD of notch 1 receptor, Invasion, Metastasis, Angiogenesis, pS473-Akt, β*-*catenin, MMP-9, VEGF, c-Myc, Bcl-2 ↑ miR-200a, Apoptosis, GSK3β, Nur77, E-cadherin	[205]
PPAR	In vitro	LIM 1899	Clofibrate	↑ Cell proliferation	[206]
	In vitro	SW620, Caco2	15d-PGJ2	↓ COX-2, VEGF, JNK pathway	[207]
	In vivo	AOM/DSS induced CRC in F344 rats	Troglitazone	↓ Aberrant crypt foci	[208]
	In vitro	MOSER	Rosiglitazone, GW7845	↑ PPAR*γ*, CEA-dependent aggregation	[209]
				↓ G1 phase, Cell number	
	In vivo	C57BL/6 J mice (AOM/DSS)	RS5444	↓ Tumor growth	[210]
	In vitro	HT-29	Rosiglitazone, 15-d-PGJ2	↓ Cell growth, ↑ Apoptosis	[211]
	In vivo	SW620 xenograft mice	Indomethacin treatment + 5-FU	↓ Tumor growth, PROM 1, CD44, PTGS2, HES1 ↑ PPAR*γ*	[212]
	In vitro	SW620	Indomethacin, Sulindac, Aspirin	↓ Colonosphere formation	[212]
	In vitro	SW620, Caco2	Indomethacin	↓ PROM1 (CD133)^+^ CD44^+^ cells	[212]
	In vitro	SW620	Celecoxib	↓ Colonosphere formation	[212]
	In vitro	SW620, Caco2	Indomethacin, 5-FU, PGE-2	↑ PROM1 (CD133)^+^ CD44^+^ cells	[212]
	In vivo	F344 rats (AOM/DSS)	Bitter melon oil (*Momordica charantia*)	↑ PPAR*γ* ↓ Tumor growth	[213]
	In vitro	HT-29	Curcumin	↑ Apoptosis, Caspase-3	[214]
				↓ PPARδ, 14–3-3ε, VEGF, β-Catenin/Tcf-4	
	In vitro	SW620, HCT-116	Troglitazone	↓ NF-κB DNA binding activity, Nuclear translocation of p50, p65, Cell growth, S phase cells, cyclin B1, cyclin D1, cyclin E, Cdk2, Cdk4, Cdc2, Bcl-2, GSK-3*β*	[215]
				↑ G0/G1 phase cell cycle arrest, Bax, Cleaved caspase-3 -9	
PPAR*δ*	In vivo	Xenograft KM12C	RNAi- PPAR*δ*	↑ Tumor growth, Tumor volume, VEGF	[216]
				↓ Differentiation, ADRP, L-FABP, ALPI	
	In vivo	Xenograft KM12C	GW501516	↓ VEGF	[216]
PPAR*α*	In vivo	Human PPAR*α* transgenic mice induced with CRC	Fenofibrate	↓ RBP1, p21, p27; adenocarcinoma, Tumor multiplicity,	[110]
				DNMT1, PRMT6	
	In vitro	HCA7	Methylclofenapate	↓ Cell number	[217]
	In vitro	SW1116 and its HCPT-resistant variant SW1116/HCPT cells	wy-14643	↓ Cell proliferation, Cell vitality	[218]
				↑ Apoptosis	
	In vitro	SW1116 and its HCPT-resistant variant SW1116/HCPT cells	MK 886	↓ Cell proliferation, Cell vitality	[218]
				↑ Apoptosis	
PPAR* β*/δ	In vitro	HT-29	NaB	↑ PPAR*β*	[219]
	In vitro	SW480	GW501516	↑ PPAR*β*/δ activity, Glut1, SLC1A5	[220]
	In vivo	AOM/DSS injected C57BL/6 wild-type mice	GW501516	↑ CRC development, Tumor number, Tumor size, PCNA, PPAR* β*/δ activity, Glut1, SLC1A5, COX-2, IL-6, IL-8, MCP-1	[220]
	In vivo	PPAR*β*^+/+^ mice induced carcinogenesis by Azoxymethane	GW0742	↓ Colon polyp multiplicity	[109]
	In vivo	PPAR*β*^+/+^ mice induced carcinogenesis by Azoxymethane	Wy-14,643	↑ ADRP, FABP	[109]
	In vivo	PPAR*β*^+/+^ mice induced carcinogenesis by Azoxymethane	Troglitazone	↑ ADRP, FABP, keratin 20, KLF4	[109]
PPAR*γ*	In vivo	Colitis induced by TNBS	Probiotics	↑ PPAR*γ*	[165]
				↓ TNF-*α*, IL-6, IFN-*γ*	
	In vitro	HT-29	Ciglitazone, 9-cis-RA	↑ Apoptosis, DNA fragmentation	[221]
				↓ COX-2, c-Myc	
	In vitro	SW480	*α*-tocopherol or *γ*-tocopherol, Troglitazone	↑ PPAR*γ*	[222]
	In vitro	HT-29	Thiazolidinedione (TZD)	↓ Cell growth, Metastasis	[223]
				↑ p21^Waf−1^, Drg-1, E-cadherin, G1 arrest	
	In vivo	Nude mice (HT-29) xenografts	TZD	↓ Tumor growth	[223]
	In vitro	SW480, HCT15, HT-29	BA, CN-BA and CN-BA-Me	↓ Cell growth	[224]
				↑ Caveolin-1, KLF4	
	Clinical	Colon adenocarcinomas tissues	–	↑ p-IκB-*α*, CBP, c-FOS, pc-Jun, EGF-R	[115]
				↓ PPAR *γ*	
	In vivo	Colon cancer tissues	–	↓ c-Myc, PCNA, PPAR *α*	[111]
				↑ Bcl-2 or Bcl-XL, PPAR *γ*	
	In vitro	SW480, LS174T	15-d-PGJ2 and pioglitazone	↓ Cell proliferation, S phase	[225]
				↑ G1 phase, TIMP‐1	
	In vitro	LS174T	15-d-PGJ2 and pioglitazone	↓ MMP-7, Invasion	[225]
	In vitro	HT-29	15-d-PGJ2 and pioglitazone	↑ Cell growth, *β*-catenin, c-Myc	[226]
	In vivo	Nude mice (HT-29) xenografts	Pioglitazone	↑ Tumor volume, *β*-catenin, c-Myc, Ki-67	[226]
	In vitro	HCT-116-XIAP^+/+^ and HCT-116-XIAP^−/−^	Rosiglitazone	↓ Cell growth, Ki-67	[227]
				↑ Apoptosis	
	In vitro	HCT-116-XIAP^+/+^	Rosiglitazone	↑ PPAR*γ*	[227]
	In vitro	HCT-116-XIAP^−/−^	Rosiglitazone	↑ PTEN	[227]
	In vivo	Balb/c nude mice (HCT-116-XIAP^+/+^ and HCT-116-XIAP^−/−^) xenografts	Rosiglitazone	↓ Tumor weight, Tumor volume	[227]
				↑ PTEN	
	In vitro	HCT-116-XIAP^+/+^ and HCT-116-XIAP^−/−^ cells	Troglitazone and 15-d-PGJ2	↓ Cell growth	[116]
				↑ Cleaved caspase-7, -8, PARP	
	In vivo	Balb/c nude mice (HCT-116-XIAP^−/−^ cells) xenografts	Troglitazone	↓ Tumor growth, Ki-67, VEGF	[116]
				↑ Apoptosis, E-cadherin	
	In vitro	HT-29-Cl.16E, Caco2, SW1116, LS174T	GW7845	↑ GPA33, p21, K19	[228]
				↓ cyclin D1	
	In vitro	DLD-1 (PSF Knockdown)	–	↑ Vacuolation, Apoptosis, Caspase-3	[229]
				↓ Cell proliferation	
	In vitro	HT-29, HCT-116	NaBt or NaBt + DHA	↑ Caspase-3, Apoptosis, Autophagy	[230]
	In vitro	HT-29 **(**PPAR*γ* siRNA**)**	NaBt or NaBt + DHA	↑ Cleaved caspase-9, -3	[230]
				↓ ALP activity	
	In vitro	HT-29, COLO 205	Ciglitazone	↑ Apoptosis	[231]
				↓ PPAR*γ*2, Cell growth	
	In vitro	HCT-116	Docosahexaenoic acid (DHA)	↓ PPAR, CD36	[232]
	In vivo	AOM-induced colon cancer model in BALB/c mice	Pioglitazone (PIO),/Rosiglitazone (RGZ),/Troglitazone (Tro)	↓ ACF formation	[233]
	In vitro	Caco2	15-deoxy-∆12, 14-prostaglandin J2	↑ PPAR*γ*	[117]
	In vitro	HT-29	Amorfrutin C	↓ Cell proliferation	[234]
				↑ Apoptosis	
	In vitro	HT-29, HCT-116	6-shogaol	↓ Cell growth	[118]
	In vitro	HT-29	6-shogaol	↑ Apoptosis, PPAR*γ,* Bax	[118]
				↓ p65, Bcl-2	
	In vitro	SW480, SW620	Linoleic acid (LA)	↓ Cell viability, Proliferation	[235]
	In vitro	HCA7, HCT-116	6-OH-11-O- hydroxyphenantrene, ciglitazone, pioglitazone	↑ TIMP1, TIMP2, Apoptosis	[236]
				↓Cell proliferation, COX-2, MMP-2, MMP-9	
PXR	In vitro	HT-29	miR-148a	↓ DNMT1, PXR, FGF-19, ALDH1A1, ABCG2, CYP3A4, Tumorspheres, CD44	[237]
	In vivo	Xenograft mice (HT-29 cells overexpressing miR-148a)	–	↓ CSC chemoresistance, Tumor recurrence	[237]
	In vivo	NOD-SCID mice (HT-29 miR-148a overexpressing cells) Xenograft	5FU + SN38	↓ Tumor growth, Sphere formation, Cell survival	[237]
	In vivo	NOD-SCID mice (HT-29 miR-148a overexpressing cells) Xenograft (PXR-Knockout)	shPXR + FOLFIRI	↓ Tumor size, Sphere formation, chemotherapy-induced enrichment of PXR, CSC markers	[238]
	In vivo	Nude mice (HT-29 cells transfected with PXR)	–	↓ Tumor size, Tumor weight, E2F1	[119]
				↑ p21^WAF1/CIP1^	
	In vitro	PXR-HT-29	–	↓ Cell proliferation, Cell viability, Colony formation	[119]
				↑ G0/G1 cell-cycle arrest	
	In vivo	Male PXR-humanized null mice (AOM/DSS)	Rifaximin	↓ Tumor number, Tumor size, Tumor incidence, iNOS, IL6, IL10, NF-κB, Blc2, c-Myc, Cdk4, Cdk6, Ccnd2, Ccna2, Cdk2, Cdk4	[239]
				↑ p21, Bcl-x, Apoptosis	
	In vitro	LS180	Budesonide	↑ CYP3A4, PXR	[240]
	In vivo	DSS-Induced Colitis Model in hPXR Mice	Rifaximin	↑ Survival rates and recovery from colitis symptoms	[241]
				↓ NF-κB	
	In vitro	HT-29, Caco2	Rifaximin	↓ NF-κB	[241]
	In vitro	LS174T	Baicalein	↑ PXR, Cdx2	[242]
	In vivo	Pxr ^+/+^ mice (DSS treated)	Baicalein	↑ PXR, Cdx2, MDR1, CYP3A11	[242]
	In vitro	LS174T	Fucoxanthin	↓ Basal and Attenuated Rifampin-Induced CYP3A4, MDR1	[243]
	In vitro	Caco2	Rifaximin	↓ Cell proliferation, Migration, VEGF, MMP2, MMP9, VEGFR-2, iNOS, p-Akt, p-mTOR, p-p70S6K, HIF-1*α*, p-p38MAPK, NF-*κ*B binding activity	[244]
	In vitro	LS180	Doxorubicin	↓ Cell viability	[245]
	In vitro	LS174T	Rifampicin	↑ PXR, SP1, MRP3	[121]
	In vitro	LS174T (after PXR activation by rifampicin)	Oxaliplatin, 5-FU	↑ Chemoresistance	[121]
	In vitro	LS174T (with PXR knocked down)	Oxaliplatin, 5-FU	↓ Cell viability	[121]
	In vitro	Caco2, HT-29, HCT-116 and SW48	5-aza-2′-deoxycytidine (5-aza-dC)	↑ CYP3A4, PXR	[246]
PR	In vitro	COLO-205	Org 31710 + Folic acid	↓ FA induced cell growth, FA-induced activations of cSrc, ERK1/2, p53, p21, p27	[247]
		COLO-205	KD of PR (Antisense oligonucleotide)	↓ FA-induced cSrc activation, p53, p21, p27	[247]
		COLO-205	FA or P4	↑ PR phosphorylation	[247]
	In vitro	COLO-205, HT-29, LoVo	FA	↓ Proliferation	[247]
	In vivo	Sprague–Dawley rats induced cancer by 1,2-dimethylhydrazine	E2 + P4	↑ Mitotic index, Apoptosis	[160]
	Clinical	CRC tissues	–	High levels of PGR expression were associated with tumor size, differentiation, vascular invasion, Tumor stage Low PGR expression led to poor prognosis	[123]
	In vitro	LoVo, SW620	Progesterone	↓ Cell proliferation, Bcl-2	[123]
				↑ CCNA1, CCNB1, Apoptosis, Cleaved caspase-3, G2/M phase cell cycle arrest	
	In vivo	Nude mice (SW620) xenografts	Progesterone	↓ Tumor volume, Tumor weight, Ki-67	[123]
				↑ TUNEL- positive cells	
RXR*α*	In vitro	mutant RARα T82A/S260-transfected Caco2 cells	9‐cisRA, cig + PD98059	↓ c-Jun, COX-2, AP-1 promoter activity, Cell growth	[124]
				↑ PPRE promoter activity, Apoptosis	
	In vitro	Moser	Bexarotene + Rosiglitazone	↓ Growth inhibition, COX-2, PGE 2, ↑ CEA	[248]
	In vivo	athymic nude mice (Moser cell xenograft)	Bexarotene + Rosiglitazone	↓ Tumor growth	[248]
	In vitro	HT-29, SW480	ATRA	↓ Cell proliferation, Sphere formation,	[125]
				ALDH + cells ↑ NSE, CgA expression	
	In vitro	HCT-116, WiDr, SW 620	ATRA (Retinol)	↓* β*-catenin	[249]
	In vitro	YAMC Transfected with DR-1Luc	DHA	↑ DR1	[250]
	In vivo	F344 rats induced cancer with Azoxymethane	Bexarotene, Bexarotene + Raloxifene	↓ Proliferation, cyclinD1, *β*-catenin, Tumor cell growth	[94]
				↑ Apoptosis, p21	
	In vitro	HCT 116	*β*-Ionone	↑ Apoptosis, G1/S phase, arrest, RXR*α* mRNA	[126]
				↓ Cell growth, Proliferation	
	In vivo	Apc^Min/+^ mice	Bexarotene	↑ RXR*α* mRNA	[251]
				↓ COX-2, PCNA, TNF*-α*, IL-1*β*, Tumor growth, cyclin D, Inflammatory cytokines	
	In vitro + In vivo	KM12C + Nude mice (BALB/c, KM12C)	Berberine	↑ p21	[252]
				↓ Proliferation, *β*-catenin, Cell growth, tumor volume, EGFR, NF-κB	
	In vitro + In vivo	KM12C, HCT-116, SW620 + KMC12C Nude mice	3,9-dimethoxy-5,6- dihydroisoquinolino[3,2-a]isoquinolin-7-ium chloride	↓ Wnt/*β*-catenin, Tumor growth, Cell growth	[253]
	In vitro	HCT-116, SW48, HT-29, SW480	Epigallocatechin-3-gallate (EGCG)	↑ RXR *α*, cell cycle arrest G1/S phase	[127]
				↓ Cell proliferation, *β*-catenin, cyclin D1, DNMT activity (in HCT-116 cells)	
	In vitro	HT-29, LoVo	Sodium valproate, 6-OH-11- Ohydroxyphenanthr -ene	↑ Apoptosis, RXR*γ*, Caspase-3, -9, Bax	[254]
				↓ Migration, cell growth, Bcl-2, HDAC1	
	In vitro	HT-29	Sodium valproate, 6-OH-11- Ohydroxyphenanthr -ene	↑ TIMP1, TIMP2	[254]
				↓ Invasiveness of cell growth, MMP9, MMP-2	
	In vitro	HCA7, HCT-116	6-OH-11-O- hydroxyphenantrene, ciglitazone, pioglitazone	↑ TIMP1, TIMP2, Apoptosis	[236]
				↓ Cell growth, COX-2, MMP-2, MMP-9	
	In vitro	HCT-116	T0901317	↑ Cell death	[188]
RXR*β*	In vitro	HCT-116	miR-22	↑ Apoptosis	[196]
THR	In vitro	Endogenous wild-type *β*-catenin in SW480 cells	T3/TR*β*1	↓ cyclin D1	[255]
	In vitro	CT26 and SW480	GC-1	↓ Cell viability	[256]
				↑ G1 cell cycle arrest	
	In vivo	CT26 xenograft mice	GC-1	↓Tumor growth	[256]
VDR	In vitro	SW480	1,25(OH)2D3	↓ Proliferation, HIF-1*α* Protein expression (Hypoxic cond.)	[257]
				↑ (VEGF, Glut-1, ET-1) (Hypoxic cond.)	
	In vitro	SW480-ADH	1,25(OH)2D3	↑ Id1	[258]
				↓ Id2, *β*-catenin, Proliferation	
	In vivo	AOM/DSS induced CRC in C57BL/6 J mice (Diet vitamin D deficient)	–	↑ COX-2, iNOS, TNF*-α*,	[132]
				Snail1, Snail2	
				↓ 25(OH)-Vitamin D/VDR	
	In vitro	LS180	1,25(OH)2D3	↓ Proliferation	[259]
				↑ c-FOS, c-Jun, CCND1, CDH1, AXIN2	
	In vivo	Intestinal VDR conditional KO (VDR^*Δ*IEC^) mice Induced CRC by AOM/DSS	–	↑ Jak2, STAT3 signaling, PCNA, Tumor	[131]
	In vivo	BALB/c mice	*L. acidophilus* *B. bifidium*	↓ Triglycerides, Alkaline, phosphatase, LDL, VDR, LPR	[260]
	In vitro	SW480-ADH	Vitamin D (3)	↑ E-cadherin, Differentiation, ZO-1	[261]
				↓ *β*-catenin, c-Myc, Tcf-1, CD44, PPAR δ	
	In vivo	VDR null mice	Vitamin D (3)	↓ Apoptosis	[262]
	In vivo	VDR null mutant mice (KO)	–	↑ PCNA, cyclin D1, Proliferation, 8-OHdG	[263]
	In vivo	Vitamin D deficient IL-10 KO mice (C57BL/6)	Calcium and 1 alpha, 25-dihydroxy vitamin D3	↓TNF-*α*, IBD	[264]
	In vivo	Wistar rats	1alpha-hydroxy vitamin D3 and 1,25-dihydroxyvitamin D3	↓ Angiogenesis	[265]
	In vitro	HT-29, DLD-1	1,25(OH)2D3	↓ H-19, c-Myc	[266]
				↑ Mad-1	
	In vivo	Nude mice	1,25(OH)2D3	↓ H19, Tumor growth	[266]
	In vivo	Nude mice	H19 (OE)	↑ Tumor growth, Induced Resistance to 1,25(OH)2D3	[266]
	In vitro	HT-29	25-hydroxyvitamin D3, 1,25(OH)2D3	↓ Proliferation	[136]
	In vivo	VDR^−/−^ mice (C57BL/6)	VDR knockout	↑ Tumor growth	[267]
				↓ Claudin-5	
	In vivo	(C57BL/6 mice)	VDR OE	↓ Tumor growth	[267]
				↑ Claudin-5	
	In vivo	Vdr^+/−^ and Apc^min/+^ mice	VDR knockout	↑ *β*-catenin, Tcf genes	[268]
	In vitro	SW480-ADH (VDR Knockdown)	1,25(OH)2D3	↑ β-catenin/TCF transcriptional activity	[268]
				↓ Inhibitory effect of 1,25(OH)2D3 on Wnt/*β*-catenin pathway	
	In vitro	SW480, HCT-116	ZnCl_2_ + 1,25(OH)2D3	↑ MT1A, MT2A	[269]
				↓ CDH1	
	In vitro	HT-29	1,25-dihydroxyvitamin D3 (1,25D3)	↑ CYP3A4	[270]
	In vitro	Caco2	1,25D and LCA	↓ *β*-catenin	[271]
	In vitro	Caco2	1,25D	↓ DKK4	[271]
	In vitro	HCT-116, Caco2, LS174T, HT-29	Snail2 OE	↓ VDR gene promoter	[137]
	In vitro	SW480-ADH	1,25(OH)2D3 + Snail2 OE	↓ Basal and 1,25(OH)2D3-induced VDR and E-cadherin expression, ligand-induced VDR transcriptional activity, 1,25(OH)2D3 effects on gene expression and Wnt/*β*-catenin pathway	[137]
	In vitro	Caco2	Curcumin	↑ VDR, VDRE, CYP3A4, CYP24, p21, TRPV6, Migration	[272]
	In vitro	HCT-116 (K-ras-mutated human colon cancer cells)	p38 MAPK activation	↑ Cell death, AP-1-dependent Trans-suppression of VDR Gene Expression	[273]
	In vitro	Caco2	1,25(OH)2D3	↑ TCF-4	[274]

**Fig. 4:**
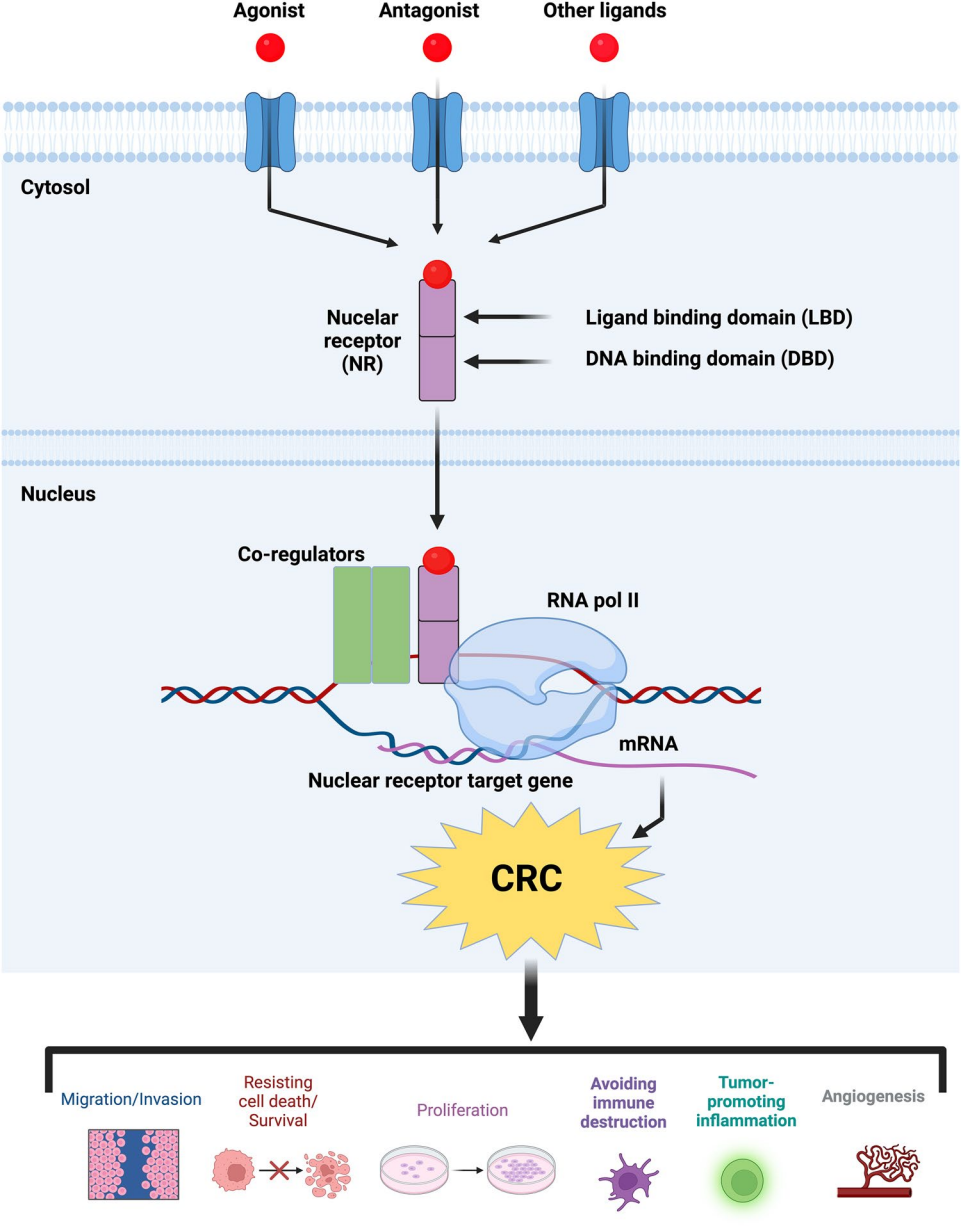
General overview of NR signaling mechanism on how various agonists, antagonist, and other ligands play a role in CRC

NRs also play a major role in the regulation of gut microbiota. Secondary bile acids (BAs) produced by the gut microbiota have a significant impact on human metabolism and energy balance via nuclear or G protein-coupled receptors. Recent research has demonstrated that BAs regulation by these receptors play an essential role in sustaining innate immune responses [68]. It is well known that the aberrant gut microbiome dysbiosis is associated with metabolic diseases including obesity, non-alcoholic fatty liver disease and insulin resistance [69]. For instance, mice models containing an intestine-specific deletion of ER*β* when administered with AOM/DSS resulted in the reduction of gut microbiota diversity. This dysbiosis induced by the synergistic effects of AOM/DSS and ER*β* deletion, further impacted cellular motility and carbohydrate metabolism, suggesting that intestinal ER*β* contributes to microbiome homeostasis, potentially reducing the risk of developing CRC [70]. The following section describes the role of NRs and their mechanistic action in CRC (Fig. 4).

### Androgen receptor (AR)

ARs, commonly referred as nuclear receptor subfamily 3, group C, gene 4 (NR3C4), belong to the NR superfamily that are ligand-dependent TFs [275, 276]. ARs consist of three major functional domains—DBD, NTD, and LBD [277]. They are primarily expressed in the kidney, testis, epididymis, seminal vesical, cervix, fallopian tube, endometrium, and breast tissues (The Human Protein Atlas: https://www.proteinatlas.org/ENSG00000169083-AR/tissue) [278]. Androgens are essential for the regulation of cell growth and differentiation in several CRC tissues. The majority of cancers, at the time of initial diagnosis, exhibit a dependence on androgens. Consequently, the primary therapeutic strategy typically involves androgen ablation therapy. This approach is designed to decrease serum androgen levels and inhibit the activity of the AR, targeting the primary drivers of tumor growth in these cases [279]. A plethora of studies have reported the pivotal role of AR in the initiation and advancement of various types of cancers [50, 280–284]. Moreover, studies in animal models suggest that androgens function as promoters in the development of colon cancer [142, 285]. Studies have elucidated the relation between AR and Wnt signaling pathway in prostate cancer. It has been evinced that AR activation is through various cofactors, such as *β*-catenin, glucocorticoid receptor interacting protein-1 (GRIP1), etc. [286, 287]. Yang and group studied the interaction and crosstalk between the AR and Wnt/*β*-catenin signaling in prostate cancer. It was reported that *β*-catenin exhibited strong and selective interaction with AR and not with other NRs like ER*α*, PR, and GR. Further, the armadillo repeats of *β*-catenin were found to directly interact with the LBD of AR [288]. Another study has reported that the translocation of *β*-catenin is facilitated by binding with AR, hence activating the Wnt signaling [289]. In another study, it was shown that limiting levels of *β*-catenin leads to AR-mediated suppression of *β*-catenin/TCF-related transcription and had no effect on AR target gene expression [290]. Also, various studies have linked the co-activation of Src and AR to be crucial in prostate tumorigenesis [291–293]. Another study reported that the Src mutant (Y527F), which displayed constitutive activity, promoted the nuclear translocation of AR and enhanced AR activity even in the absence of androgens. Conversely, the Src mutant with inactive kinase activity demonstrated a downregulation of AR transcriptional activity [291]**.** Hence the interplay between the AR, Src and *β*-catenin orchestrates the transcriptional activation of AR.

It was noted that the genes encoding the AR consists of 2 polymorphic trinucleotide repeat segments, which are polyglutamine (CAG) and polyglycine (GGC). These CAG repeats are inversely related to numerous cancers [294]. Since AR is expressed in colon tissues, alterations in the length of the CAG repeat of the AR gene can also be associated with colon cancer [294]. Further, it was observed that AR and VDR signaling are interlinked, and they work mutually in CRC [295]. For example, individuals with 23 or more CAG repeats of the AR gene, less exposure to sunlight, and low intake of vitamin D tend to show an increased rate of rectal carcinoma development, mainly amongst men when compared to women [295]. Multiple studies have reported the downregulation of AR in CRC tissues when compared to adjacent normal tissues [71, 73, 74, 296]. However, its expression was also reported to be upregulated in CRC tissues implicating the differential role of AR in CRC development and progression [72].

**Fig. 5:**
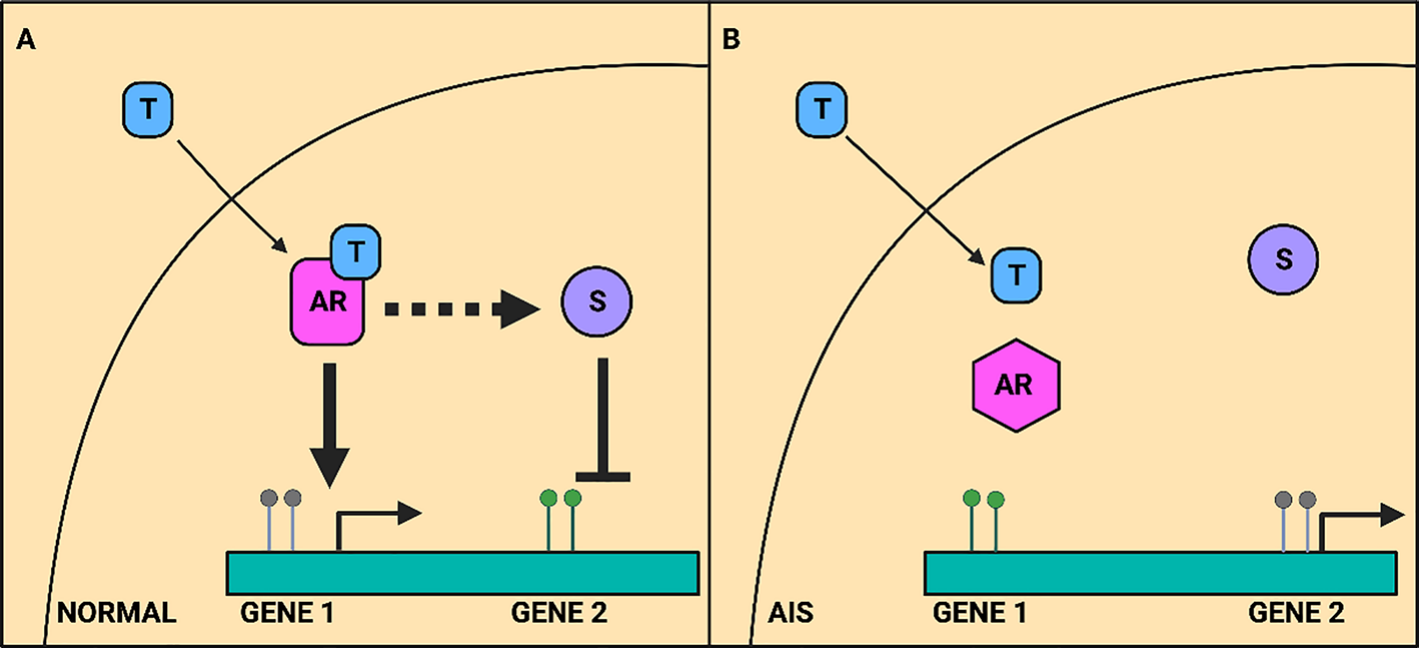
Activity of AR in DNA methylation- **A** Nonmutant, inactive androgen receptor binds to testosterone resulting in receptor activation. Androgen receptor response elements are bound with the activated androgen receptor causing DNA-inducing gene expression and preventing DNA methylation. Additionally, suppressor complexes (S; dotted arrow) that repress specific gene sets (“Gene 2”) are affected by either the active AR itself or AR-induced genes, which ultimately results in the DNA methylation of suppressed genes. **B** In Androgen insensitivity syndrome, the absence of AR activity leads to the inhibition of AR target genes, which may cause (stochastic de novo) DNA methylation of the affected genes. On the other hand, genes that AR typically silences (directly or through additional AR-dependent pathways) are activated, which prevents DNA methylation. Grey lollipops: unmethylated DNA, Greens lollipops: methylated DNA

Several studies suggested the drugs that modulate the expression of AR have remarkable potential in the prevention and treatment of CRC. For example, FCX, an arylidene derivative, suppressed the cell growth of AR-selective HCT-8 and HT-29 colon cancer cell lines in higher FCX concentrations [139]. In another study, it was observed that dehydroepiandrosterone (DHEA) and nerve growth factor (NGF) decreased, serum deprivation-induced apoptosis, but the treatment with testosterone led to increased apoptosis in colon cancer cell line (Caco2), suggesting the interplay between steroid hormones and neurotrophins signaling in hormone-dependent tumors [50]. Xia et al., elucidated the role of AR gene methylation in the modulation of CRC (Fig. 5) [297]. Another study revealed that enzalutamide, an AR antagonist, enhanced the myeloid cell-mediated immune suppression and progression in both in vitro and in vivo [140]. Further, treatment of SW480 cells with DHT in combination with casodex effectively disrupted the androgen-sensitive interaction between AR and *β*-catenin, and concurrently alleviated the transcriptional repression of the TOPFLASH reporter. Furthermore, an increase in the accumulation of cells at the G1 phase of the cell cycle was observed. Concurrently, in vitro growth assays demonstrated a 35% decrease in the viability of cells treated with the AR+DHT [141]. In another study, the combination of selective androgen receptor modulator (SARM), GTx-024, with histone deacetylase inhibitor (HDACi), AR-42, was found to improve anabolic response in the cachectic condition in CRC by mediating the regulation of *β*-catenin in C-26 cachectic mice model [142]. Therefore, AR and its modulators play a key role in regulating different processes involved in CRC and hence could be an important target in the treatment and clinical management of CRC.

### EAR 2

EAR 2, also known as nuclear receptor subfamily 2, group F, gene 6 (NR2F6), is an orphan NR belonging to the member of the chick ovalbumin upstream promoter-transcription factors (COUP-TFs) that regulate various biological processes like migration, adhesion, apoptosis, etc. [75, 298]. The expression of EAR 2 is highly upregulated in human primary colorectal tumors when compared to normal colon tissues. It was observed that the knockdown of EAR 2 in CRC cell lines HCT-116, RKO, and HT-29 resulted in the inhibition of X-linked inhibitor of apoptosis protein (XIAP) expression and induction of apoptosis. Further, in the same study, the EAR 2-inactivated RKO xenograft model showed suppression of tumor growth, thus suggesting the role of EAR 2 in regulating cell survival in colon cancer [75]. However, more studies are needed to establish the potential of EAR 2 as a therapeutic target for CRC.

### Estrogen receptors (ERs)

The ER, a steroid hormone NR, acts as a TF and governs the expression of target genes implicated in diverse processes, including cellular proliferation and survival [299]. Following the activation of the receptor, ER dimerizes when they are in contact with a ligand [300]. The ligand estradiol (E2) activates ER resulting in the dimerization, nuclear translocation and biding to the RE of the target gene that is located in or adjacent to the promoter regions [301]. The ER is categorized into two groups, namely estrogen receptors alpha (ER*α*) and beta (ER*β*), which are mainly involved in regulating multiple physiological processes in the human body [302]. This receptor has been reported to play a significant role in modulating a variety of pathological disorders, including cancer [303, 304].

A plethora of studies have shown the association of ER with CRC, and it is variably expressed in this cancer [76–80, 82–92, 94, 100]. Moreover, both isoforms are reported to have different functions in CRC. For instance, ER*β* was reported to exhibit a protective effect in CRC through its activation by estrogen [305]. It was observed that ER*β* exhibited contrasting results of p65 chromatin binding in HT-29 and SW480 cells. In HT-29 cells, ER*β* diminished a significant portion of p65 chromatin binding, whereas in SW480 cells, it augmented p65 binding. This could be due to the appearance of new p65 binding sites in SW480 cells in the presence of ER*β*, resulting in distinct modulation of the p65 cistrome in both cell lines [305].

Hartman et al., demonstrated that ER*β* has the ability to impede cell proliferation and suppress tumor growth in both in vitro and in vivo, likely due to the inhibitory effects of ER*β* on cell-cycle pathways. Moreover, this repression of the cell cycle by ER*β* relies on the functional binding with estrogen response elements (EREs) [86]. Studies have shown that low expression of ER induces colon carcinogenesis and its progression; however, overexpression of this receptor inhibited cell viability and induced apoptosis by upregulating Bax, p53, and cleaved caspase -3 and -9 [81, 149]. Moreover, the differential expression of ER*α* and ER*β* was found to regulate numerous miRNAs, signaling pathways like Wnt/*β* catenin, p38/MAPK, etc., and induce apoptosis [81, 149, 154, 155, 157–159]. An intriguing study reported that over-expression of ER*α* inhibited cell proliferation and induced apoptosis by upregulating the expression of the hTNF*-α* gene and downregulating *β*-catenin signaling in LoVo cells. The same study has also shown the overexpression of hER*α* and E2 treatment enhanced the promoter activity of TNF-*α* in these cells [148]. Moreover, it was shown that the deficiency of ER*β* increased small intestine tumorigenesis in murine models and was correlated with the modulation of genes implicated in TGF*β* signaling with or without estrogen treatment [154]. Another study suggested that the treatment of cisplatin with SW480 ER*β* cells resulted in the increased cell viability [163]. In addition, ER*β* knockout mouse model was found to develop higher colitis-associated colon carcinogenesis [93]. The downregulated ER*β* expression also led to higher inflammatory damage caused by upregulating TNF*-α* and nuclear factor-κB (NF-κB) target molecules [93]. Furthermore, the upregulation of ER*β* was found to increase miRNA-205 levels in both normal and cancerous colon epithelial cells, subsequently reducing PROX1 expression, leading to decreased proliferative and metastatic potential of the cells [95]. Contrastingly, ER*β* was also shown to exhibit tumor-promoting effect. For instance, it was shown that ER*β* was positively correlated with colon carcinogenesis in a rat model; however, the treatment with ER*β* antagonist, raloxifene, inhibited aberrant crypt foci (ACF) formation in this model [146].

A plethora of studies have also identified the potential of ER agonists and antagonists modulating the activity of ER. For example, activation of ER*β* with agonist ERB-041 in HCT-116, Caco2, and SW480 cell lines decreased cell survival, colonosphere formation, and migration while increasing the expression of ESR2, HPGD, CCND1, CTNNB1, CSLTR1, etc., suggesting the anti-tumor role of ER*β* in CRC and the possible use of its agonist in the treatment of this disease [147]. Moreover, 17-*β* estradiol and progesterone increased the expression of ER*β,* which led to elevated apoptosis by decreasing proliferating cell nuclear antigen (PCNA) and upregulating the expression of caspase -3 and -8 with enhanced cleavage of Poly (ADP-ribose) polymerases (PARP) in the experimental model [160]. The modulators of ER, such as tamoxifen and raloxifene, were also shown to reduce cell growth, and proliferation in HCT-116 and HCT-8 cell lines [143]. In another study, tamoxifen and 5-Flurouracil (5-FU) alone, or in combination inhibited cell migration, proliferation and induced apoptosis and cell cycle arrest with downregulation of ER*β* and matrix metalloproteinase 7 (MMP-7) in HT-29 colon cancer cells [156]. In addition, the agents like celecoxib and difluoromethylornithine (DFMO) was found to exhibit chemopreventive effect by modulating ER*α* expression and DNA methylation [80]. Further, it was shown that various natural compounds and other agents like 5-Aza-CdR, quercetin, curcumin, ginseng, raloxifene, soy isoflavones, folic acid, genistein, resveratrol, and silymarin exhibit anti-cancer activity by modulating the expression of ER [146, 153, 162, 306, 307]. With regard to this, the induction of ER*β* by dietary soy isoflavones demonstrated an anti-cancer effect by suppressing cell growth and tumor dysplasia in both in vitro experiments using DLD-1 cells and in a rat model [153]. In another study, the activation of ER*β* with apigenin and naringenin showed cancer-preventive effects in young adult mouse colonocytes (YAMC) cells [144]. Moreover, 5-Aza-CdR induced the expression of ER*α* and ER*β* via the downregulation of DNMT1, which resulted in apoptosis and inhibition of cell growth in HT-29 CRC cells [162]. Thus, ER could be a potential target, and modulating this NR with various agonists/antagonists and other agents holds immense prospects for the management of CRC.

### Estrogen related receptor (ERR)

The ERR, also known as nuclear receptor subfamily 3, group B (NR3B), is one of the orphan receptors belonging to the NR superfamily of ligand-regulated TFs, which significantly regulates the cellular metabolism of the body [308]. It is structurally more related to the canonical ER and can modulate estrogen signaling in most types of cancers [308]. The presence of ERR mainly in the metabolically active tissue regions helps in regulating the transcription of metabolic genes, consisting of the ones involved in mitochondrial turnover and autophagy [309]. ERRs are classified into three isoforms, among which ERR*α* and ERR*β* were first cloned in 1988 by using the DBD of ER*α* as the probe to screen recombinant DNA libraries [310]. Later, the third isoform ERR*γ* was identified [310]. Vernier et al., reported a direct molecular connection between the activity of two isoforms of ERR and the regulation of glutamine utilization as well as the production of the antioxidant glutathione. The downregulation of ERR*α* limits the entry of glutamine into the TCA cycle, whereas the upregulation of ERR*γ* enhances the production of glutathione driven by glutamine. Significantly, it was also observed that elevated expression of ERR*γ* serves as a notable indicator of oxidative stress induced by mitochondrial dysfunction or chemotherapy [310]. Recent evidence have reported the critical role of ERR in various metabolic diseases and cancer [309, 311]. In line with this, studies have also evaluated the role of ERR in regulating various molecules and processes involved in CRC [96, 97, 99, 312].

The expression of ERR*α* was found to be upregulated in CRC cells and tissues [96, 97, 99, 312]. In addition, ERR*α* overexpression was associated with shorter OS and progression-free survival (PFS) and correlated with advanced stage and higher tumor grade [99]. Additionally, the overexpression of ERR*α* was found to increase the proliferation and migration of CRC cells via elevating the expression of IL-8 [312]. However, numerous studies suggested that inhibiting ERR*α* significantly impaired cell proliferation, migration, colony formation, cell cycle arrest, and induced apoptosis by downregulating several metabolic pathways and associated molecules [164]. For example, inhibition of ERR*α* induced cell cycle arrest, apoptosis, and increased mitochondrial metabolic stresses, ROS generation, and mitochondrial membrane permeabilization [98]. Moreover, the inhibition of ERR*α* decreased the expression of peroxisome proliferator-activated receptor gamma coactivator 1-alpha (PGC-1*α*), cytochrome c oxidase subunit IV, and voltage-dependent anion channel 1 (VDAC1) and reduced mitochondrial oxidative phosphorylation (mtOxPhos), mitochondrial DNA (mtDNA) copy number and intracellular ATP levels in HCT-116 p53^+/+^ cells [98]. In addition, the knockdown of ERR*α* and inhibition of its expression with agents like XCT790 significantly inhibited cell growth and colony formation by reducing c-Myc and cyclin D1 expression in HCT-116 and SW480 CRC cell lines [97]. Further treatment with trametinib, a specific MEK inhibitor, suppressed cell growth by reducing the expression of ERR*α* and its downstream molecule IDH3A. Furthermore, the combined treatment of trametinib and simvastatin, resulted in the suppression of ERR*α* transcriptional activity, culminating in a synergistic impact on the inhibition of proliferation and survival of colon cancer cells in pre-clinical settings [97]. Additionally, it was shown that osteopontin (OPN), an oncogene involved in tumor progression, is a direct target of ERR*α* and thus, silencing of ERR*α* resulted in a marked reduction of OPN at both protein and RNA levels in HT-29 cells, suggesting the significance of targeting ERR*α* in CRC [96]. Collectively, these studies suggest that ERR*α* is a potential target for the treatment and management of CRC.

### Farnesoid X-receptor (FXR)

FXR also known as nuclear receptor subfamily 1, group H, gene 4 (NR1H4) is a well-characterized member of the metabolic subfamily of NRs [276, 313]. It is mainly expressed in the liver and intestine, and due to its pivotal role in regulating BA homeostasis, it is also referred to as BA receptor [314]. Upon binding of ligands, the FXR regulates the function of essential genes that are implicated in the metabolism of lipids and carbohydrates. Therefore, due to these important functions, it is considered one of the most promising drug targets for the treatment of BA-related liver diseases [315, 316]. Obeticholic acid (OCA) is approved as the first small molecule to target FXR, and various other small molecules are being evaluated in clinical trials [317, 318]. As ligands for FXR, bile acids, oxysterols, and cholestanoic acid take part in the complex web of interactions that ultimately control the lipid, steroid, and cholesterol homeostasis [313]. It has been shown that FXR has been linked to distinct roles specific to certain tissues and cells within different cancer types. Further, FXR has exhibited its ability to modulate a multitude of cellular signaling pathways, encompassing NF-κB, EGFR/ERK, JAK/STAT PI3K/Akt, Wnt/*β*-catenin, and p38/MAPK, along with their respective targets. These targets consists of a diverse array of molecules, such as EMT markers, MMPs, caspases, tumor suppressor proteins (such as C/EBP*β*, p-Rb, and p53), cyclins, various cytokines, and numerous other entities. As a result, FXR exhibits potential as a cutting-edge target for the identification, prognosis, and treatment of cancer [43, 166, 168, 174]**.** According to several studies, the rate of cancer cell proliferation and tumor aggressiveness were linked with the overexpression of FXR in different cancers of breast, esophageal, lung, pancreas, and thyroid [43, 319–321].

Many studies have revealed both tumor-suppressive and oncogenic roles of FXR in CRC [35, 42, 43, 100, 322]. The FXR expression increased with the degree of differentiation in HT-29 and Caco2 cells, and it was also demonstrated that the FXR was downregulated in colon carcinomas and adenomas [101]. Further, FXR and PPAR*γ* expression was inversely correlated in CRC [101]. Mao et al., demonstrated that silencing of FXR by small interfering RNAs (siRNAs) resulted in the Wnt/*β*-catenin signaling activation and formation of *β*-catenin/TCF4 complex in HT-29, Caco2 and HCT-116 cells [166]. FXR activation with agonists, like CDCA and GW4064, resulted in the dose-dependent suppression of MMP-7 in HT-29 cells. Additionally, it was observed that there was an increase in protein and mRNA levels of MMP-7 in the intestinal tissues and liver homogenates in the FXR knockout mice (B6.129X1 (FVB)-Nr1h4^tm1Goz^/J) [174]. In the same study, FXR overexpression was also shown to suppress MMP-7 expression and cell invasion in MC38 cells [174]. Another study showed that FXR overexpression and treatment with the agonist GW4064 suppressed CRC cell proliferation by inhibiting p-EGFR (Tyr845), p-ERK, and p-Src. On the other hand, inhibition of FXR by siRNA or guggulsterone induced p-EGFR (Tyr845) and p-ERK leading to increased cell proliferation in CRC. Moreover, the upregulation of FXR suppressed CRC tumor growth in nude mice, implying the role of Src, FXR, and EGFR in intestinal cell proliferation and tumorigenesis [168]. Selmin et al., observed the impairment of APC function favors the knockdown of FXR expression via CpG hypermethylation in both murine colonic mucosa and human colon cells. This downregulation led to a decreased expression of downstream targets such as SHP and IBABP, which are critical for BA homeostasis. Concurrently, there was an upregulation of pro-inflammatory and oncogenic factors like COX-2 and c-Myc, contributing to the pathogenesis of colon carcinogenesis [171]. Moreover, it was shown that FXR deficiency increased CRC cell proliferation by upregulating the expression level of cyclin D1, IL-6 and increased size and multiplicity of small intestine adenocarcinomas in CRC mouse models [167]. In addition, it was shown that chenodeoxycholic acid binds to FXR and upregulates the miR-22 expression, inhibiting cyclin A2 (CCNA2) and inducing G0/G1 cell cycle arrest in HCT-116 cells. Additionally, FXR knockout mice exhibited downregulation of miR-22, upregulation of ileal CCNA2 and increased Ki67-positive cells in the colon [169]. In another study, it was observed that activation of FXR by GW4064 led to the upregulation of death receptor 5 (DR5), and reduced cell proliferation upon treatment with TRAIL and GW4064 in CRC cell lines (HCT-116, SW480, DLD-1) [170]. In addition, it was found that FXR activation by GW4064 in colon cancer cell lines, SW620 and HCT-116 upregulated the expression of cyclin G2 (CCNG2) by suppressing miR-135A1, which leads to reduced cell proliferation and induction of cell cycle arrest. However, the knockdown of FXR reversed this effect by upregulating the expression of miR-135A1 and suppressing CCNG2 [172]. Another study revealed that FXR knockout mice (FXR^−/−^Apc ^Min/+^) showed a reduction in survival rate and an increase in size and number of AOM/DSS-induced colon tumors revealing the significant potential of FXR in suppressing colorectal carcinogenesis [173]. Therefore, understanding the tumor suppressive role of FXR in CRC and thus modulating its expression by agonists and antagonists might be helpful in the management of colon tumorigenesis.

### Hepatocyte nuclear factor 4 alpha (HNF4*α*)

The ligand-dependent TF HNF4*α,* is also known as nuclear receptor subfamily 2, group A, gene 1 (NR2A1) [276]. It is a highly conserved member of the NR superfamily and is expressed in both liver and gastrointestinal tract. In the liver, HNF4*α* is mainly known for its role as the master regulator of liver-specific gene expression and is vital for both fetal and adult liver functioning [276, 323]. The dysregulation of HNF4*α* expression has been linked with various human diseases like colon cancer, hepatocellular carcinoma, liver cirrhosis, ulcerative colitis, and maturity-onset diabetes of the young [323, 324]. HNF4*α* is linked to numerous signaling pathways that significantly contribute to tumor transformation, metastasis, inhibition of apoptosis, and promotion of proliferation. Lv et al., demonstrated that HNF4*α* participates in the aberrant activation of various signaling pathways, including the NF-κB pathway, Wnt/*β*-catenin pathway, and STAT3 pathway. Its involvement in these pathways plays an essential role in the initiation and advancement of cancer, including CRC [325]**.** The dysregulation of the Wnt/*β*-catenin signaling pathway plays a role in several cancer types. Wu et al., reported that the upregulation of HNF4*α* can inhibit tumor progression by suppressing the Wnt/*β*-catenin signaling pathway [326]**.** HNF4*α* plays a key role in the modulation of NF-κB signaling during cancer development. It promotes the expression of interleukin 1 receptor type 1 (IL1R1) and subsequently enhances the inflammatory response triggered by its ligand, interleukin 1*β* (IL1*β*). The activation of NF-κB signaling by IL1*β*/IL1R1 leads to the upregulation of HNF4*α*, establishing a feedback loop that sustains NF-κB pathway activation and propels inflammation towards cancer development [327]**.** The association between STAT family proteins and human carcinoma has been extensively established, and the constitutive activation of STAT3 plays a pivotal role in the process of carcinogenesis [328]**.** Further, HNF4*α* can disrupt the regulation of miR-122, leading to the upregulation of c-Met and subsequent activation of STAT3 [329]. Therefore, HNF4*α* has the ability to reverse tumor lesions by inhibiting the activation of the STAT3 signaling pathway and suppressing the invasion and metastasis of cancer cells. It has been observed that loss of HNF4*α* affects ion transport and induces chronic inflammation like inflammatory bowel disease in mice [330]. Moreover, it was shown that the inactivation of HNF4*α* in colon cancer cells and conditionally knockout mice decreased the expression of the gut-specific homeotic TF Cdx2, suggesting their positive correlation and tumor suppressive activity of HNF4*α* in colon cancer [331]. In addition, it was shown that ectopic overexpression of HNF4*α* suppressed proliferation, migration, invasion, and promoted G2/M phase arrest and apoptosis in HT-29, SW480, and LoVo cells. Besides, overexpression of HNF4*α* inhibited EMT via modulating Wnt/*β*-catenin signaling and suppressing the expression of Snail, Slug, Twist, and Vimentin while inducing E-cadherin expression in colon cancer cells. The same study also showed that the overexpression of HNF4*α* suppressed tumor growth and liver metastasis in SW480 xenograft model [102]. Contrastingly, studies have also identified the tumor-promoting activity of HNF4*α.* For example*,* a study by Xu et al., showed that the increased expression of HNF4*α* induced by the overexpression of tumor-promoting lncRNA LINC00858, resulted in suppression of WNK lysine deficient protein kinase 2 (WNK2) and progression of carcinogenesis in colon cancer cells [175]. In addition, lectins such as lens culinaris agglutinin (LCA) was shown to promote CRC by inducing the gene expression of HNF4*α* along with other genes like glucose-6-phosphatase (G6Pase) and phosphoenolpyruvate carboxykinase (PEPCK) in Caco2 cells [332]. Additionally, it was found that the inactivation of HNF4*α* inhibited cell proliferation and differentiation by blocking the gene expression of MUC4 and PCNA in HM7 cells [177]. Further, studies reported the divergent roles of HNF4*α* isoforms in tumorigenesis. Specifically, the HNF4*α*8 isoform was shown to promote tumor progression by enhancing cellular proliferation, invasion, and migration. In contrast, the HNF4*α*2 isoform exhibited tumor-suppressive properties, as evidenced in both in vitro and in vivo experimental models [176, 333]. Thus, these studies suggest the differential function of HNF4*α* in CRC. However, more studies are required to have a better understanding of its role in this cancer.

### Liver receptor homolog 1 (LRH-1)

LRH-1, a member of the nuclear receptor 5, group A, gene 2 (NR5A2) subfamily is expressed in the tissue regions derived from the endodermal origin, including the exocrine pancreas, intestine, liver, ovary, placental region, and pre-adipocytes [334, 335]. They are predominantly regulated by cofactor interactions [336]. They modulate various functions like tissue-specific cell proliferation, cholesterol homeostasis, steroidogenesis, and stem cell pluripotency [334]. LRH-1 plays a major role in various biological processes such as gastrulation, differentiation, development, maintaining reverse cholesterol transport, BA, and glucose homeostasis [334–336]. It was reported that LRH-1 was involved in etiology of various tumor types, encompassing breast, gastric, pancreatic and colon cancer [334]. Several studies showed that LRH-1 is highly upregulated in colon cancer patients and is associated with poor OS, which might suggest LRH-1 as a beneficial prognostic molecular marker for the treatment of CRC [103–105]. For example, in a clinical study, the expression of LRH-1 was investigated in 128 cases of colon cancer, alongside their adjacent normal tissues using immunohistochemistry. The results revealed positive LRH-1 expression in 108 out of 128 colon cancer samples, while only 17 out of 128 adjacent normal tissues showed LRH-1 expression. The statistical analysis demonstrated a significant association between positive LRH-1 expression and various clinical pathological factors, including stage, depth of invasion, and lymph node metastasis. Patients with high LRH-1 expression had a notably lower OS rate compared to those with low expression. Furthermore, the multivariate analysis indicated that LRH-1 expression could serve as an independent predictor of OS. Overall, the observations of this study signify that LRH-1 likely plays a crucial role in the onset and advancement of CRC. It has the potential to serve as a valuable prognostic molecular marker, offering assistance in the diagnosis of colon cancer [103]**.** Moreover, it was shown that LRH-1 plays a significant role in intestinal tumorigenesis by regulating cell cycle and inflammatory proteins such as cyclin D1, cyclin E1, c-Myc, and TNF-*α* in animal model [184, 334]. A study by Lai et al., has shown the association of LRH-1 in promoting cancer stemness by acting as a direct target of GATA6 and elevating the levels of stem cell markers such as ALDH‐1, Ascl2, CD133, CD44, and LGR5 in CRC cells. Overexpression of LRH-1 also leads to the induction of HIF‐1*α* and its target genes, resulting in stronger glycolysis and lactate accumulation in HCT-116 and HT-29 clones [178]. Moreover, overexpression of LRH-1 was shown to increase the expression of steroidogenic enzymes and cortisol synthesis while its downregulation inhibited these processes implying the novel mechanism of tumor immune escape via glucocorticoids synthesis in colon cancer [337, 338]. In addition, it was shown that LRH-1 modulates the expression of PPAR*γ* by maintaining the synthesis of cortisol in Caco2 cells [185]. Additionally, the knockdown of LRH-1 leads to cell cycle arrest and suppression of cell proliferation via Wnt signaling cascade in Caco2 and HT-29 cells [179]. LRH-1 was also shown to promote CRC cell growth by suppressing the CDKN1A gene expression mediated through p53 pathway [186]. Further, the downregulated expression of miR-381 and miR-30d led to the modulation of its direct target, LRH-1 resulting in the induction of proliferation and invasion in CRC cells [104, 181]. Furthermore, miR-136 and miR-374b was found to suppress the proliferation and invasion of CRC cells by targeting LRH-1 and Wnt signaling [105, 180]. Thus, these studies highlight the importance of targeting LRH-1 as a potential target for combating CRC.

### Liver X receptors (LXRs)

LXRs, an oxysterols receptor, have two isoforms: LXR*α* and LXR*β*, which belongs to the NR superfamily and play crucial roles in the transcriptional regulation of lipid metabolism [339, 340]. LXRs bind to the genes and regulate their expression that encode proteins involved in cholesterol efflux, absorption, excretion, transport, and conversion of BA in the liver [341]. As LXRs are involved in controlling membrane structures and functions, they also provide novel therapeutic insights into the pathophysiology of diseases like diabetes mellitus, atherosclerosis, and cancer that are linked to dysregulated lipid metabolism [339]. Studies have revealed that LXRs are involved in the progression of inflammatory diseases of the nervous, cardiovascular, and respiratory systems, and therefore targeting this receptor could result in the inhibition of these diseases including cancer [340, 341].

Interestingly, LXRs activation has been reported to affect cell survival and cell proliferation of various types of cancers that distort the metabolic pathways resulting in the accumulation of cholesterol [342]. Lin et al., stated the potential efficacy of LXRs’ ligands in the management of cancer [343]. In line with this, the role of LXR in CRC was reported by many studies and revealed that LXR was diversely expressed in this disease [106, 191]. However, Tang et al., found that the expression level of LXR was downregulated in CRC patients [189]. Moreover, the inactivation of LXR in mice model showed increased tumorigenesis by inducing the expression of proliferation markers, while treatment with its agonist GW3965 inhibited this effect. Furthermore, the activation of LXR was shown to induce cell cycle arrest by modulating the expression of genes such as, Skp2, c-Myc, CDKs, cyclins, SCD1, p15 and hypo-phosphorylation of the retinoblastoma (Rb) tumor suppressor protein [191]. Additionally in another study, the activation of LXR was found to induce G1 phase arrest and caspase-dependent apoptosis in vitro and also suppressed colon cancer tumor growth in a mouse model [106].

Further, activation of LXR*β* was shown to induce cell death by modulating the expression of NLRP3, caspase-1, -3, -7, -8, and -9 as confirmed by preclinical studies [190, 344]. In another study, it was shown that cytoplasmic localization of LXR*β* promoted the ligand induced pyroptosis in colon cancer cells, while this process was not observed in normal colon epithelial cells [188]. It was also demonstrated that mice treated with LXR agonist T0901317 induced immunogenic cell death by modulating the levels of calreticulin, HMGB1, and ATP in CT26 cells [194]. The activation of LXR by its agonist, T0901317, was found to suppress the proliferation, clonogenicity, and migration by upregulating ABC transporters, ABCA1, ABCG5, and ABCG8, in HT-29 CD133^+^ cells. Similarly, its antagonist, SR9243, also showed inhibition in the proliferation and clonogenic potential of these cells by increasing ROS generation and suppressing metabolic enzymes such as PFKFB3, GSK3*β*, FASN, and SCD-1, thus suggesting the role of LXR in regulating cancer stem cells, colon tumorigenesis, and metastasis [187]. Moreover, treatment of HCT-116 cells with LXR agonists LXR623 and GW3965 along with ABT263 and BH3 mimetics induced the expression of LXR*β,* which subsequently led to elevated levels of apoptosis and inhibition of cell viability, thus proposing the potential of LXR agonists and BH3 mimetics to be plausible agents for the treatment of solid malignancies [193]. Further, a clinical study with 37 patients have elucidated that the expression of LXR was downregulated in tumor tissues. Another clinical study with 707 patients has revealed that positive expression of LXR and COUP-TFII were observed in 50.9% (360/707) and 32.7% (231/707) of the CRC tissues, respectively. However, it was noted that the presence of LXR and COUP-TFII expression exhibited an association with improved OS rates. Therefore, the expression of LXR and COUP-TFII, in combination, may serve as biomarkers indicating positive prognosis in patients diagnosed with CRC [346]**.** Overall, preclinical studies have demonstrated that LXR agonists have shown promise for the treatment of CRC. Further clinical investigation is needed to assess the safety and efficacy of LXR agonists as plausible therapeutic agents in this disease. Therefore, clinical trials involving LXR agonists as part of combination treatment regimens for CRC are ongoing, suggesting the promising efficacy of LXR and its agonists/antagonists for the treatment and management of CRC.

### Nuclear hormone receptor 77 (Nur77)

Nur77, commonly known as nerve growth factor-induced B alpha (NGFI-B *α*), NR4A1, NGFIB, TR3, TIS1, NAK-1, or N10, is an orphan NR, which is known for its endogenous ligands [107, 347]. The Nur77 act as an immediate early response gene that is initiated through multiple signal transduction mechanisms [348]. It plays a vital role in cell differentiation, proliferation, and apoptosis [348]. Nur77 has been found to function as both tumor and anti-tumor gene in CRC depending on its cellular context [349, 350]. Studies have also evaluated the expression and role of Nur77 in different cancers, including CRC. For instance, the NR Nur77 was found to be highly expressed in colon tumors and was found to induce survival in CRC cells [107, 108]. Moreover, induction of Nur77 by its agonist, deoxycholic acid (DCA), was found to induce cell growth, colony formation, and migration by modulating Wnt/*β*-catenin and AP-1 pathways and upregulating BRE and VEGF in CRC cells [108]. In addition, it was observed that hypoxia induced the expression of Nur77, which subsequently increased *β*-catenin via PI3K/Akt signaling and was found to augment cell migration, invasion, and EMT in CRC cells [198]. Additionally, the activation of Nur77 with BA was found to regulate genes involved in cell survival and apoptosis, such as CDK4, CCND2, MAP4K5, STAT5A, RBBP8, and Bid in colon cancer cells [202]. On the other hand, studies have also reported the anti-tumorigenic role of Nur77 in CRC. For instance, Nur77 was shown to promote the proteasomal degradation of an oncogenic protein, *β*-catenin, in SW620 colon cancer cells, thereby suggesting the tumor-repressive property of this NR in CRC [351]. Niu et al., revealed that Nur77’s role in colon cancer is specifically defined by its effects on inhibitor of differentiation 1 (ID1), a target gene of TGF*β*, expression and is modulated by the potency of the TGF*β* signal. Nur77 suppresses ID1 expression to function as a tumor suppressor in a low TGF*β*-signal environment, whereas, Nur77 promotes the growth of tumors by enhancing the effect of TGF*β* on the induction of ID1 under situations of strong TGF*β* signal [350]**.** Further, agents like indomethacin, sulindac, and 5-FU were found to activate Nur77, which led to apoptosis in CRC cells [200, 352]. In addition, miR-22 was found to upregulate the expression of Nur77 and RAR*β* and suppress HDAC, leading to increased apoptosis in CRC cells. In the same study, the activation of miR-22, Nur77, and RAR*β* and reduction of HDACs were found to supress tumor growth in CRC xenograft [196]. Furthermore, multiple studies have demonstrated the anti-cancer efficacy of Nur77 agonist, 1,1-bis(3′-indolyl)-1-(phenyl)methane(DIM-C-Ph), 1,1-bis(3′-indolyl)-1-(p-anisyl)methane (DIM-C-pPhOCH3), in CRC cells. It was found to inhibit cell growth, survival, migration, and invasion while inducing apoptosis by elevating the expression of TRAIL, PARP, PDCD1, CSE, ATF3, and CSE and activating caspases -3, -8, and -9 [107, 197, 199, 201].

Moreover, another orphan NR, Nurr1, also known as nuclear receptor 4 group A, gene 2 (NR4A2), belongs to the same NR subfamily of Nur77 [276]. Nurr1 is expressed ubiquitously throughout the body and grouped under gene 2 (NR4A2). It is a transcriptional regulator that is crucial for the formation, maintenance, and differentiation of meso-diencephalic dopaminergic (mdDA) neurons [278, 348, 353]. It is necessary for the transcription of a group of genes, including SLC6A3, SLC18A2, tyrosine hydroxylase, and DRD2, whose expression is required for the growth of mdDA neurons [353]. NR4A2 further serves as a significant and critical junction linking the eicosanoid and fatty acid metabolic pathways through its transcriptional integration. Moreover, it was also observed that induction of NR4A2 by PGE2 resulted in binding to the Nur77-binding response element located within the peroxisome proliferator response element, activating the fatty acid oxidation genes, including FABP2, FABP4, ACOX, and CPT1M. Thus, PGE2 can be used to regulate the shift toward fatty acid oxidation, which is observed in several types of cancer, to control energy utilization [204]. Nurr1 was also reported to play a pivotal role in the development and progression of CRC [348]. Another study by Holla et al., reported that activation of Nurr1 by PGE2 promoted cell survival by inducing fatty acid oxidation and its associated proteins in LS-174 T and HCT-116 CRC cells. Thus, this study suggests the role of Nurr1 in regulating the process of ATP generation in CRC cells [204]. The precise mechanism by which Nur77 acts as an oncogene and tumor suppressor gene in CRC is still not well understood. Further investigation is warranted to better understand the intricate roles of Nur77 and Nurr1 in the development of colorectal tumors, and to develop therapeutic strategies that can be used to target these genes in order to improve the clinical outcomes of CRC patients.

### Progesterone receptor (PR)

PR, identified as nuclear receptor subfamily 3, group C, gene 3 (NR3C3), is a ligand-dependent transcription factor belonging to the NR family. Its primary function involves the regulation of target gene expression through binding with its specific steroid hormone ligand, progesterone (P4). Further, PR serves as a central controller in processes such as proliferation, differentiation, and development, particularly during the reproductive cycle and pregnancy in female reproductive tissues [354, 355]. Studies have implicated a potential association between the PR levels and the risk of CRC. For instance, various studies have indicated an upregulation of PR expression in CRC tissues compared to normal mucosa [76, 122]. In another study, Zhang et al., proposed that decreased expression of PR and its ligand, P4, correlates with an unfavorable prognosis in CRC patients. Treatment of P4 inhibited cell proliferation, induced cell cycle arrest, and promoted apoptosis. These effects were mediated through the activation of the JNK pathway via DNA damage-inducible protein *α* (GADD45*α*), leading to the suppression of malignant progression in CRC. Additionally, P4 treatment resulted in reduced tumor volume and weight in CRC xenografts, suggesting a potential inhibitory role for P4 and PR in improving the prognosis of CRC patients [123]. Further, treatment with E2 and P4 induced apoptosis by enhancing the expression of PCNA and upregulating cleaved PARP, caspase-3, and cleaved caspase-8 levels in vivo [160]. Furthermore, folic acid (FA) treatment significantly attenuated the proliferation rate of PR-positive COLO-205, HT-29, and LoVo cells by activating c-Src and inducing the expression of cell cycle regulators p21, p27, and p53. However, this effect was nullified by pre-treatment with a PR-specific antagonist, Org 31710, highlighting the involvement of PR in FA-mediated inhibition of proliferation [247]. Taken together, PR emerges as a pivotal NR in regulating mechanisms associated with cell growth, proliferation, and apoptosis in the context of CRC.

### Peroxisome proliferator-activated receptors (PPARs)

The PPARs belong to the NR superfamily, which plays a crucial function in lipid and glucose metabolism and acts as a ligand-inducible TF [356]. PPARs are present in 3 isoforms—PPAR*α*, PPAR*β*/*δ*, and PPAR*γ* and are classified as nuclear receptor subfamily 1, group C (PPAR*α*-NR1C1; PPAR*β*/*δ*-NR1C2; PPAR*γ*-NR1C3) in the current nomenclature system [356, 357]. The three PPARs are variably expressed in various tissues [358]. PPAR*β*/*δ* is more widespread; however, it is mostly found in skin, skeletal muscle, heart, adipose tissue, liver, and inflammatory cells. PPARγ comprises three distinct variant isoforms (*γ*1, *γ*2, and *γ*3) with different tissue localization [358]. All the isoforms of the PPARs form heterodimers with RXR to either activate or repress the downstream target genes. They are known to regulate multiple conditions, such as hypertension, inflammation, and atherosclerosis [356]. Further, due to the special role of PPAR*β*/*δ,* this receptor is known as an important therapeutic target for various disorders, including cancer [359]. Furthermore, it has also been observed that designing agonists of PPARs, might improve its therapeutic values in cancer [356]. Studies have reported the activation/suppression of PPAR*β* and PPAR*δ* expression in various cancer cell models have resulted in the modulation of CRC [111, 117, 360]. In several studies, it was exhibited that PPARs were highly upregulated in CRC models [111–114, 116–118]. Contrastingly, few studies have also reported the downregulation of PPARs in CRC [109, 110, 115]. PPAR activation has been shown to decrease cell growth as well as trigger differentiation and apoptosis in a range of cancer cell types [361–363]. With regard to this, combined treatment of indomethacin with 5-FU significantly reduced tumor growth by activating PPAR*γ* and suppressing the expression of PROM 1, CD44, PTGS2, and HES1 in SW620 xenograft mice [212]. The treatment of PPAR ligand, rosiglitazone, was found to suppress tumor growth in HCT-116-XIAP^(−/−)^ xenograft model via the upregulation of PTEN [227]. Moreover, PPAR*γ* ligand thiazolidinedione (TZD) was demonstrated to suppress cell growth, metastasis and induce G1 phase arrest by upregulating the expression of p21^Waf-1^, Drg-1, and E-cadherin and reducing tyrosine phosphorylation of *β*-catenin in HT-29 cells. Further, TZD treatment was also found to block lymph nodes and lung metastasis in xenograft mice [223]. In addition, the treatment of pioglitazone and 15-deoxy-delta (12,14)-prostaglandin J2 (15-d Δ PGJ2) blocked the proliferation, invasion and initiated G1 phase cell cycle arrest by suppressing MMP-7 and elevating TIMP-1 level in SW480 and LS174T cell lines [225]. Another study demonstrated that the overexpression of miR-506 in an HCPT-resistant colorectal carcinoma cell line contributed to the resistance against HCPT by suppressing PPAR*α* expression. These findings offer a scientific basis for formulating miRNA-centered therapeutic approaches to counter drug resistance in HCPT-resistant CRC [218]. The induction of PPAR*γ* by bitter melon oil (BMO) (*Momordica charantia*) was also found to suppress tumor growth in a rat model [213]. Another PPAR agonist, troglitazone, was found to induce apoptosis and G0/G1 cell cycle arrest by attenuating the expression of NF-κB, and GSK-3*β* in SW620 and HCT-116 cells [215]. Additionally, amorfrutin C, a PPAR*γ* agonist having low affinity for PPAR*γ*, was shown to inhibit cell proliferation and induce apoptosis by disrupting mitochondrial integrity and inducing caspases, DNA fragmentation, PARP cleavage, externalization of phosphatidylserine, and ROS generation in HT-29 cells [234]. Further, the activation of PPAR*γ* with 6-shogaol initiated apoptotic cell death by suppressing the activity of NF-κB in HT-29 cells [118]. Furthermore, Mielczarek-Puta et al., showed that linoleic acid (LA), a PPAR*γ* agonist, decreased the cell viability and proliferation in SW480 and SW620 cells in a concentration-dependent manner [235]. It was also found that silencing of PPAR*δ* increased cell proliferation by enhancing the expression of VEGF in KM12C cells while this effect was reversed by bevacizumab, a specific VEGF inhibitor [216]. Moreover, troglitazone treatment was found to repress cell growth in various colon cancer cells in vitro [364]. Additionally, the treatment of several agonists such as troglitazone, pioglitazone, and rosiglitazone, suppresses tumor growth and ACF formation in mice, suggesting a possible involvement of PPAR at the initial phase of CRC development [233]. Further, the treatment with GW0742, Wy-14,643, and troglitazone induced markers involved in colonocyte differentiation, along with other markers such as ADRP, FABP, keratin 20, and KLF4 in PPAR*β*^+/+^ mouse model [109]. Contrastingly, it was reported that the low dosage of PPAR*γ* ligand 15-d Δ PGJ2 and pioglitazone promoted cell growth and tumor growth in APC-mutated HT-29 cells and its xenograft mouse model, respectively, by elevating the expression of c-Myc, and *β*-catenin [226]. Moreover, Zou et al., has reported that GW501516, a PPAR*δ* agonist, increased the expression of Glut1 and SLC1A5 in SW480 cells and enhanced the colitis-associated CRC by inducing pro-inflammatory genes, such as COX-2, IL-6, IL-8, and MCP-1 in AOM/DSS-exposed mice [220].

Several clinical trials targeting PPARα and PPARγ receptors are currently ongoing for the treatment of different cancers [http://clinicaltrials.gov/]. In a Phase I, multicenter clinical trial, combinatorial treatment of efatutazone, a selective PPAR-*γ* agonist and paclitaxel was found to be safe and well tolerable in 15 patients of anaplastic thyroid cancer. Moreover, it was found that angiopoietin-like 4 was induced by the treatment of efatutazone in the biopsy samples of patients [365]. Another dose escalated clinical study determined the effectiveness of efatutazone in solid malignancies patients. It was observed that 0.5 mg twice daily was safe and induced a sustained partial response in a patient with myxoid liposarcoma. Moreover, the treatment also showed stable disease (SD) response for more than 60 days in 10 patients [366]**.** Komatsu and group evaluated the efficacy of efatutazone in conjunction with FOLFIRI treatment against metastatic CRC. Combinatorial treatment had significant safety profile and stable disease progression with increased levels of adiponectin in plasma [367]. Recently, a Phase I interventional clinical trial (ClinicalTrials.gov ID NCT03829436) is ongoing to explore the tolerability, safety and tumor inhibiting activity of TPST-1120, selective antagonist of PPAR*α* as monotherapy and with nivolumab, an anti-PD1 antibody against solid tumors including CRC. It is now well known that targeting PPARs could be an important approach in the battle against CRC, although, it is a long road ahead for successfully establishing PPARs in clinical settings. The major drawback for PPAR based therapy is the differential expression of the PPARs in various cancers and its context dependent response, thereby impeding the development of a universal and common PPAR agonist or antagonist for all cancers. Moreover, the off targets effects induce side effects which could be detrimental in the treatments. With the advent of advanced omics and technologies, it is prudent to develop reliable biomarkers which could help in predicting the therapy response in CRC that could pave the way for integrating personalized medicine approach in NRs based treatments. Hence these findings suggest the role of PPARs in CRC, and targeting this group of NRs with its ligands is of significant importance for the management of CRC.

### Pregnane X receptor (PXR)

PXR, also known as nuclear receptor subfamily 1, group I, gene 2 (NR1I2), is a prototypical member of the NR superfamily, which is known to be stimulated by the endobiotics and xenobiotics [368]. PXR being a key xenobiotic receptor generally binds to the regulatory gene sequences in a ligand-dependent manner [369]. Detoxification, metabolism, and inflammation are some of the common downstream targets of PXR in xenobiotic responses [368]. It was also reported that PXR signaling was involved in various biological process like proliferation, apoptosis, cell cycle arrest, angiogenesis, and oxidative stress [368]. PXR is widely expressed in both normal and malignant tissues [370]. Moreover, it was also reported that PXR has a crucial role in cancer stem cells (CSC)-mediated tumor recurrence. In addition, it was also observed that its expression in CSCs plays an important role in modulating gene expressions that are involved in chemoresistance and self-renewal [238].

Several studies have reported that PXR is variably expressed in various CRC cell lines and tissues which might regulate different processes of colon carcinogenesis [119–121]. Overexpression of miR-148a was found to suppress the expression of PXR along with DNMT1, FGF-19, ALDH1A1, ABCG2, CYP3A4, and CD44, which subsequently inhibited tumorsphere formation in HT-29 cells. Moreover, the overexpression of miR-148a also led to decreased CSC chemoresistance in HT-29 xenograft mice [237]. However, in another study, it was shown that PXR was downregulated in colon tumors and the ectopic upregulation of PXR inhibited cell proliferation and elevated G0/G1 cell cycle arrest by upregulating p21^(WAF1/CIP1)^ and reducing E2F1 expression in HT-29 cells. Moreover, the ectopic expression of PXR was found to reduce tumor size and weight in HT-29 xenograft mice [119]. Moreover, the overexpression of PXR was associated with poor recurrence-free survival in CRC patients [238]. However, in an in vivo study, it was found that the knockdown of PXR induced the chemosensitivity and ameliorated the self-renewal property of CSCs and slowed down the process of tumor recurrence in a mouse model exposed to chemotherapeutic drug [238]. In addition, several preclinical studies have demonstrated the efficacy of the chemotherapeutic drug rifaximin in inhibiting proliferation and inducing apoptosis in CRC experimental models by upregulating hPXR and suppressing the expression of various molecules such as VEGF, MMP-2, MMP-9, VEGFR-2, iNOS, p-Akt, p-mTOR, p-p70S6K, HIF-1*α*, p-p38MAPK, TNF-*α*, iNOS, IL-6, IL-10, and NF-κB [239, 241, 244]. Further, the treatment of CRC cells with rifampicin enhanced the expression of PXR, SP1, and MRP3, which suggests the role of PXR in inducing resistance to chemotherapeutic agents in CRC cells [121]. Furthermore, baicalein, a herbal flavonoid was found to activate PXR in a Cdx2-dependent manner, suggesting the potential involvement of PXR in inducing anti-inflammatory and anti-cancer activities in CRC. Baicalein treatment was also shown to reduce MDR1 and CYP3A11 expressions in PXR^+/+^ in vivo model [242]. In another study, the treatment of LS174T cells with fucoxanthin attenuated the drug resistance by suppressing rifampin-induced multiple drug resistance 1 (MDR1) and cytochrome CYP3A4 mRNA expression via PXR-mediated pathways [243]. However, a study by Zimmermann et al., showed that glucocorticoids like budesonide induced the expression of molecules involved in drug metabolisms like CYP3A4 and CYP3A11 via modulation of PXR [240]. Moreover, in a study, it was reported that methylation of the PXR promoter modulated the mRNA expression of PXR and CYP3A4 in CRC cell lines, suggesting the involvement of PXR/CYP3A4 axis during colon carcinogenesis and drug responses. However, the treatment of CRC cells, Caco2, HT-29, HCT-116, and SW480, with 5-Aza-dC, reversed the process of DNA methylation [246]. Therefore, PXR plays an important role in several processes of drug response and metabolism and could be an important target to overcome drug resistance in CRC.

### Retinoid X receptors (RXR)

RXRs, also known as nuclear receptor subfamily 2 group B (NR2B), a member of the NR superfamily, which mainly consists of three isoforms, i.e., *α*, *β*, and *γ*, are found in every cell type in humans [276, 371, 372]. It plays an important role in nutrient metabolism through heterodimerization with other NRs such as CAR, FXR, LXR, PPAR, PXR, etc. [372]. RXR was also reported to suppress cell proliferation and induce apoptosis in various cancers by its homodimerization with selective agonists or rexinoids [124, 372, 373]. Studies have shown that RXR*α* was downregulated in CRC tumorigenesis, as confirmed in CRC tissues and in vivo mouse models [126, 127]. However, some studies have also reported the upregulation of RXR in CRC and its association with the induction of other molecules involved in the RA pathway, such as ALDH1, RAR, CYP26A1, and CtBP1 [124, 125]. The different findings on the expression of RXRs in CRC can be influenced by a wide pleotropic factors including tumor stage differences, genetic backgrounds, and experimental procedures used in the investigations. These variables may contribute to the disparities and variabilites observed in RXR expression levels and functional roles in CRC. For instance, tumor stage is an important factor to consider when evaluating RXR expression in CRC. RXR expression levels may differ at different phases of CRC development and progression [126]. Some studies, for example, have found decreased RXR expression in advanced CRC stages, implying that RXRs may play a role in early tumorigenesis [126, 127]. However, few findings have showed elevated RXR expression in certain CRC cases, revealing a possible connection with tumor growth or resistance to therapeutic interventions [124, 125]. As a result, changes in tumor stage distribution between study cohorts can alter the overall conclusions about RXR expression in CRC. As it is well known that CRC being the heterogeneous disease has varied genetic alterations, RXR expression and function can be influenced by genetic factors such as mutations in important signaling pathways or transcriptional regulators. These genetic variances across patients can result in changes in RXR expression profiles [126]. The heterogeneity in experimental methodologies used to determine RXR expression may also contribute to the contradictory results. Discrepancies in the expression could occur due to differences in sample collection, RNA extraction, and quantitative methodologies for detecting RXR expression levels. Furthermore, changes in antibody specificity and sensitivity in immunohistochemistry or immunofluorescence assays can impact RXR protein expression detection and interpretation. Janakiram et al., revealed that induction of RXR with its agonist, *β*-ionone, inhibited cell proliferation and induced G1/S-phase cell cycle arrest and apoptosis in HCT-116 cells. Further, activation of RXR*α* also inhibited AOM-induced ACF in colon carcinogenesis in a rat model [126]. Similarly, a study by the same group showed that treatment with bexarotene, an RXR*α* agonist, suppressed colon tumorigenesis by inhibiting cyclin D1, COX-2, and PCNA in Apc ^(Min/+)^ mouse model. In addition, dose-dependent treatment of bexarotene also suppressed serum triglycerides and inflammatory cytokines in a mouse model [251]. Studies have also reported that berberine and its analog 3,9-dimethoxy-5,6-dihydroisoquinolino [3,2-a] isoquinolin-7-ium chloride (B-12) activate RXR*α,* which further leads to the reduction of cell growth by downregulating the Wnt/*β*-catenin pathway in CRC cells. Subsequently, it was observed that the shRXR*α* KM12C cell xenograft model treated with berberine resulted in the suppression of tumor growth [252, 253].

The phosphorylation of RXR*α* was associated with colon carcinogenesis; however, inhibition of this phosphorylation and inducing the heterodimerization of unphosphorylated RXR–PPAR*γ* in the presence of their ligands, 9-cisRA, and ciglitazone, synergistically suppressed cell growth and induced apoptosis by reducing COX-2 and c-Jun at both protein and RNA level [124]. Moreover, the combination of RXR and PPAR*γ* agonists, bexarotene and rosiglitazone respectively, inhibited cell growth by suppressing COX-2 and PGE2 while increasing carcinoembryonic antigen (CEA) in Moser CRC cells. The combination treatment was also found to suppress tumor growth in Moser cells xenograft mouse model [248]. In addition, the treatment of ATRA-resistant HCT-116, WiDr, and SW620 cells with retinol suppressed cancer cell growth by augmenting the proteasomal degradation of *β*-catenin via the RXR-mediated pathway [249]. Further, it was shown that DHA induced a chemopreventive effect against colon carcinogenesis by modulating RXR*-*PPAR axis in YAMC and NCM460 cells [250]. A bioactive component of green tea, epigallocatechin-3-gallate (EGCG), was also found to inhibit cell proliferation and induced G1/S phase cell cycle arrest by upregulating the expression of RXR*α* and suppressing *β*-catenin, cyclin D1, and DNA methyltransferase activity in CRC cell lines [127]. Moreover, the combined treatment of sodium valproate (VPA), an HDAC inhibitor, with a RXR ligand, 6-OH-11-O-hydroxyphenanthrene (IIF), was found to induce apoptosis and reduce cell viability and invasion by increasing the expression of RXR*γ* and apoptotic proteins such as Bax and cleaved caspase-3 and -9, tissue inhibitor matrix metalloproteinase 1 (TIMP1) and TIMP2 while reducing Bcl-2, MMP-2, and MMP-9 in HT-29 cells [254]. Further, the combination of IIF with ciglitazone was demonstrated to enhance apoptosis and attenuate cell growth and migration by elevating RXR*γ,* PPAR*γ*, TIMP1, and TIMP2 and inhibited the expression of COX-2, MMP-2, MMP-9 and PGE2 in HCA-7 and HCT-116 cells [236]. Hence, RXRs play a crucial role in cell proliferation and survival, thereby modulating RXRs with specific agonists or antagonists might be beneficial in the treatment and management of CRC.

### Thyroid hormone receptors (THR)

The THRs along with their isotypes THR*α*1, and THR*β*1 belong to the NR superfamily that regulates thyroid hormone signaling in various tissues to mediate numerous important developmental and physiological processes [374]. They are mainly known for their role as ligand-dependent TFs, and the THRs bind to the thyroid hormone RE irrespective of the presence or absence of thyroid hormone to modulate the expression of target genes [374]. It was observed that the expression of THR was upregulated in thyroid cancer patients [375]. In addition, the levels of THR*α*1 was also highly upregulated in human CRC patients and was found to modulate the Wnt pathway [128]. However, Horkko et al., reported that the expression of THR*β*1 was higher in normal human mucosa when compared to CRC samples. This expression study suggests that the downregulation of this NR at an advanced stage suggests its tumor-suppressive function [129]. THRs expression patterns in CRC have revealed inconsistent results in several studies, and multiple variables could contribute to these variations. Tumor stage, genetic background, and experimental procedures used in the investigations are all potential variables impacting the reported THR expression differences. For instance, THRs subtypes expression levels could differ at different stages of CRC development and progression. Some studies, for example, have found lower THR*β*1 expression in advanced CRC stages, implying that THR*β*1 may play a tumor-suppressive function in early carcinogenesis. THRs might have potential therapeutic implications in CRC treatment, particularly in terms of cell viability, cell cycle arrest, and reduction in tumor growth [128, 129]. Preclinical investigations have shown that activating THRs with thyroid hormone analogues or synthetic ligands reduces CRC cell survival. These ligands bind to THR and regulate gene expression as well as signaling pathways involved in cell proliferation, survival, and apoptosis. The downstream effects include cell cycle arrest at several checkpoints, such as the G1 and G2/M phases, as well as the stimulation of apoptosis in CRC cells. Moreover, THR activation has been shown to increase the susceptibility of cancer including CRC to conventional chemotherapeutic drugs, resulting in better treatment outcomes [376, 377]**.** THR agonists have also been studied as prospective combination therapy with other targeted medicines, such as EGFR inhibitors, to alleviate efficacy and overcome resistance mechanisms [376]. In this context, a study by Natsume and group demonstrated that the inhibition of endogenous wild type *β*-catenin with triiodothyronine (T3)/THR*β*1 in SW480 cells with mutation of the APC gene resulted in the inhibition of cyclin D1 through the Tcf/Lef-1 site [255]. Moreover, activation of THR*β*1 by its agonist, GC-1, was found to induce an anti-proliferative effect by decreasing cell viability and increasing G1 phase arrest in CT26 and SW480 cells. In the same study, treatment of GC-1 showed a tremendous reduction in tumor growth in CT26 xenograft mouse model [256]. Thus, these studies suggest the significant importance of THR in the management of CRC; however, more studies are warranted to explore the potential mechanism behind the role of this NR in CRC.

### Vitamin D receptor (VDR)

VDRs belong to the NHR superfamily, which acts as ligand-inducible TFs [378]. VDR is expressed in all tissues of the bone, breast, colon, kidney, lung, ovary, pancreas, etc. [379]. Several co-activator complexes are essential for the ligand-mediated transactivation of VDR [378]. Most of the biological processes involving vitamin D are known to be exerted through VDR and mediate processes like cell proliferation, differentiation, and calcium homeostasis [378, 379]. Studies have also shown the role of vitamin D and its receptor in cancer [379–381]. In CRC, VDR has anti-cancer properties and was found to be downregulated in CRC cells and tissues [130–133, 135, 137, 138]. However, few studies have also reported that the expression of VDR was upregulated in CRC tissues compared to normal adjacent tissues [134, 136]. Moreover, it was shown that the expression of VDR was sequentially upregulated from normal to a well-differentiated tumor while decreasing in poorly differentiated tumor [379].

VDR was first identified as a biomarker for vitamin D-mediated suppression of cell proliferation in human colon cancer [382]. VDR and its ligands play an important role in the regulation of several genes and signaling pathways linked to CRC. The activation of VDR by its ligands, such as 1,25-dihydroxyvitamin D3 (calcitriol), impacts gene expression and many cellular processes in colon cancer cells. In the colon, VDR activation has been demonstrated to decrease cell proliferation, promote differentiation, induce apoptosis, and suppress inflammation [383]**.** VDR signaling also interacts with critical pathways involved in colon cancer, such as Wnt/*β*-catenin, PI3K/Akt, and MAPK signaling. Further, several types of immune cells express VDR and are controlled by calcitriol, which may contribute to its anti-CRC activity. Given the relevance of the intestinal microbiota in CRC and the discovery that it is affected by vitamin D deficiency, an indirect antitumoral action of calcitriol at this level could be vital in CRC progression [384]**.** For instance, it was shown that 25(OH)D3, a VDR ligand, exhibited an anti-proliferative effect and induced apoptosis by regulating the expression of VDR in SW480 cells [379]. Additionally, 1,25(OH)2D3, a high-affinity VDR ligand, was known to regulate the transcription of various genes in CRC by activating VDR and modulating its binding to the target genes such as *β-*catenin [385]. Further, the AOM/DSS-induced colon carcinogenesis was associated with the inhibition of VDR and upregulation of Snail1, Snail2, COX-2 and iNOS in a murine model [132]. Furthermore, many studies have demonstrated the role of 1,25-(OH)2D3 in regulating VDR and various other genes such as TNF*-α,* CYP3A4, c-FOS, c-Jun, CCND1, Snail1, Snail2, CDH1, AXIN2, TCF-4, TCF7L2, etc. to exhibit anti-cancer property by suppressing the CRC hallmarks [259, 261, 262, 270, 274]. In another study, it was shown that the treatment of SW480 and HCT-116 cells with ZnCl_2_ induced the expression of MT1A, MT2A, and 1,25(OH) 2D3-induced cadherin 1, thus suggesting the linkage of VDR with zinc signaling in CRC [269]. Thus, VDR and its ligand play a pivotal role in regulating different signaling and molecules associated with CRC.

## Conclusion

Despite the advancement in recent research, colon cancer remains one of the most common malignancies globally. The diagnosis of CRC at an advanced stage is linked to poor prognosis and diminished survival outcomes among patients. Thus, it is necessary for the identification of biomarkers that could be a target for the management of CRC. Regarding this, NRs were known to be deregulated and differentially expressed in CRC and were shown to modulate different processes associated with this cancer. The different NRs, such as AR, EAR 2, ER, ERR, FXR, HNF4*α,* LRH-1, LXR, Nur77, PPAR, PXR, PR, RXR, THR, and VDR, were found to modulate different signaling pathways such as NF-κB, Wnt/*β*-catenin, MAPK, etc. These NRs and their modulators (agonists/antagonists) were found to regulate various proteins and genes associated with major pathways involved in CRC progression, which subsequently led to the suppression of cell survival, proliferation, migration, invasion, and induction of apoptosis in CRC. Further, several agonists and antagonists of NRs have been shown to enhance the anti-tumor efficacy through a combinatorial approach with other anti-cancer compounds, such as bexarotene and raloxifene in CRC [94, 126]. Growing lines of evidence have established that NRs targeting could be a viable treatment intervention in CRC, although this treatment modality has its own set of limitations and challenges. The intricacy of NRs signaling pathways is the major limitation in treatment, as NRs can have varied and context-dependent consequences on tumor progression and therapy response. Further, the differential expression levels and transcriptional activity of NRs amongst various cancer types, makes it improbable to develop a universal NR-targeted therapy. Furthermore, the off-target effects and acquisition of drug resistance against NR agonists and antagonists can have unanticipated results and debilitating side effects. Further research in exploring the intricate cross talk and mechanistic action of NRs is vital in developing putative targets that can be exploited for diagnosis, prognosis and therapeutic approaches for CRC. Moreover, randomized, double blind, multicentered clinical trials are necessary for establishing the safety and efficacy of these agonists and antagonists. Overall, while NRs show potential as cancer therapeutic targets, addressing these limits and obstacles are crucial for their effective and safe implementation in clinical settings for CRC. Due to this, several clinical trials are currently being carried out to investigate the potential of NR-targeting small molecules as CRC therapeutics.

## Data Availability

Not applicable.

## Abbreviations

5-oxo-ETE: 5-Oxoeicosatretraenoic acid
15-d Δ PGJ2: 15-Deoxy-delta (12,14)-prostaglandin J2
ACF: Aberrant crypt foci
Akt: Protein kinase B
ALP: Alkaline phosphatase;
AOM: Azoxymethane
AP1: Activator protein 1
APC: Adenomatous polyposis coli
AR: Androgen receptor
B-12: 3,9-Dimethoxy-5,6-dihydroisoquinolino [3,2-a] isoquinolin-7-ium chloride
BAR: Bile activated receptor
Ber: Berberine
THR: Thyroid hormone receptor
CAF: Cancer-associated fibroblasts
CAR: Constitutive androstane receptor
CDH 1: Cadherin 1
ChIP: Chromatin immunoprecipitation
CRC: Colorectal cancer
CSC: Cancer stem cells
DBD: DNA-binding domain
DHA: Docosahexaenoic acid
DKK 1: Dickkopf Wnt signaling pathway inhibitor 1
DSS: Dextran sulfate sodium
EAR2: v-Erb A related-2
ECGC: Epigallocatechin- 3-gallate
EGFR: Epidermal growth factor receptor
ER: Estrogen receptor
EREs: Estrogen-responsive elements
ERR *α*: Estrogen related receptor alpha
ERR*γ*: Estrogen related receptor gamma
ER*α*: Estrogen receptor alpha
ER*β*: Estrogen receptor beta
Ex4: Exendin 4
FGF 2: Fibroblast growth factor 2
FXR: Farnesoid X receptor
GA: Glycyrrhetinic acid
GL: Glycyrrhizin
GLP1R: Glucagon-like peptide-1 receptor
Glut1: Glucose transporter 1
HDI: Human development index
HIF-1*α*: Hypoxia-inducible factor 1-alpha
HMGB1: High mobility group box 1
HNF4*α*: Hepatocyte nuclear factor 4 alpha
HRT: Hormone-replacement therapy
JNK: C-Jun N-terminal kinases
LA: Linoleic acid
LCA: Lens culinaris agglutinin
LDHA: Lactate dehydrogenase A
lncRNAs: Long non-coding RNAs
LRH1: Liver receptor homolog 1
LXR: Liver X receptors
MCT4: Monocarboxylate transporter 4
mdDA: Meso-diencephalic dopaminergic
MEK: Mitogen-activated protein kinase kinase
miRNA: MicroRNA
MMP 7: Matrix metalloproteinase 7
mRNA: Messenger RNA
mtDNA: Mitochondrial DNA
NF: Normal fibroblasts
NF-κB: Nuclear factor kappa B
NGFI-B alpha: Nerve growth factor-induced B alpha
NR: Nuclear receptor
Nur77: Nuclear hormone receptor 77
OCA: Obeticholic acid
OPN: Osteopontin
OS: Overall survival
PCNA: Proliferating cell nuclear antigen
PDK1: 3-Phosphoinositide-dependent kinase 1
PEPCK: Phosphoenolpyruvate carboxykinase
PGC-1*α*: Peroxisome proliferator-activated receptor gamma coactivator 1-alpha
PGRMC1: Progesterone receptor membrane component 1
PI3K: Phosphoinositide 3-kinase
PPAR: Peroxisome proliferator-activated receptors
PR: Progesterone receptor
PSF: PTB-associated splicing factor
PXR: Pregnane X receptor
RAR: Retinoic acid receptor
ROS: Reactive oxygen species
RXR: Retinoid X receptors
SF 1: Steroidogenic factor-1
shRNA: Short hairpin RNA
siFXR: Fxr-small interfering RNA
TIMP: Tissue inhibitor metalloproteinase
OXER1: Oxoeicosanoid receptor 1
TCA: Tricarboxylic acid cycle
TF: Transcription factor
TGF*β*: Transforming growth factor *β*
TNF-*α*: Tumor necrosis factor alpha
THR: Thyroid Hormone Receptor
TRPM8: Transient receptor potential melastatin 8
TZD: Thiazolidinedione
UTR: Untranslated region
VDAC1: Voltage-dependent anion channel 1
VDR: Vitamin D receptor
VEGF: Vascular endothelial growth factor
